# A quantitative evaluation of computational methods to accelerate the study of alloxazine-derived electroactive compounds for energy storage

**DOI:** 10.1038/s41598-021-83605-2

**Published:** 2021-02-18

**Authors:** Qi Zhang, Abhishek Khetan, Süleyman Er

**Affiliations:** 1 DIFFER–Dutch Institute for Fundamental Energy Research, De Zaale 20, 5612 AJ Eindhoven, The Netherlands; 2 CCER–Center for Computational Energy Research, De Zaale 20, 5612 AJ Eindhoven, The Netherlands; 3 Department of Applied Physics, Eindhoven University of Technology, 5600 MB Eindhoven, The Netherlands

**Keywords:** Energy, Computational chemistry, Density functional theory

## Abstract

Alloxazines are a promising class of organic electroactive compounds for application in aqueous redox flow batteries (ARFBs), whose redox properties need to be tuned further for higher performance. High-throughput computational screening (HTCS) enables rational and time-efficient study of energy storage compounds. We compared the performance of computational chemistry methods, including the force field based molecular mechanics, semi-empirical quantum mechanics, density functional tight binding, and density functional theory, on the basis of their accuracy and computational cost in predicting the redox potentials of alloxazines. Various energy-based descriptors, including the redox reaction energies and the frontier orbital energies of the reactant and product molecules, were considered. We found that the lowest unoccupied molecular orbital (LUMO) energy of the reactant molecules is the best performing chemical descriptor for alloxazines, which is in contrast to other classes of energy storage compounds, such as quinones that we reported earlier. Notably, we present a flexible in silico approach to accelerate both the singly and the HTCS studies, therewithal considering the level of accuracy versus measured electrochemical data, which is readily applicable for the discovery of alloxazine-derived organic compounds for energy storage in ARFBs.

## Introduction

**Figure 1:**
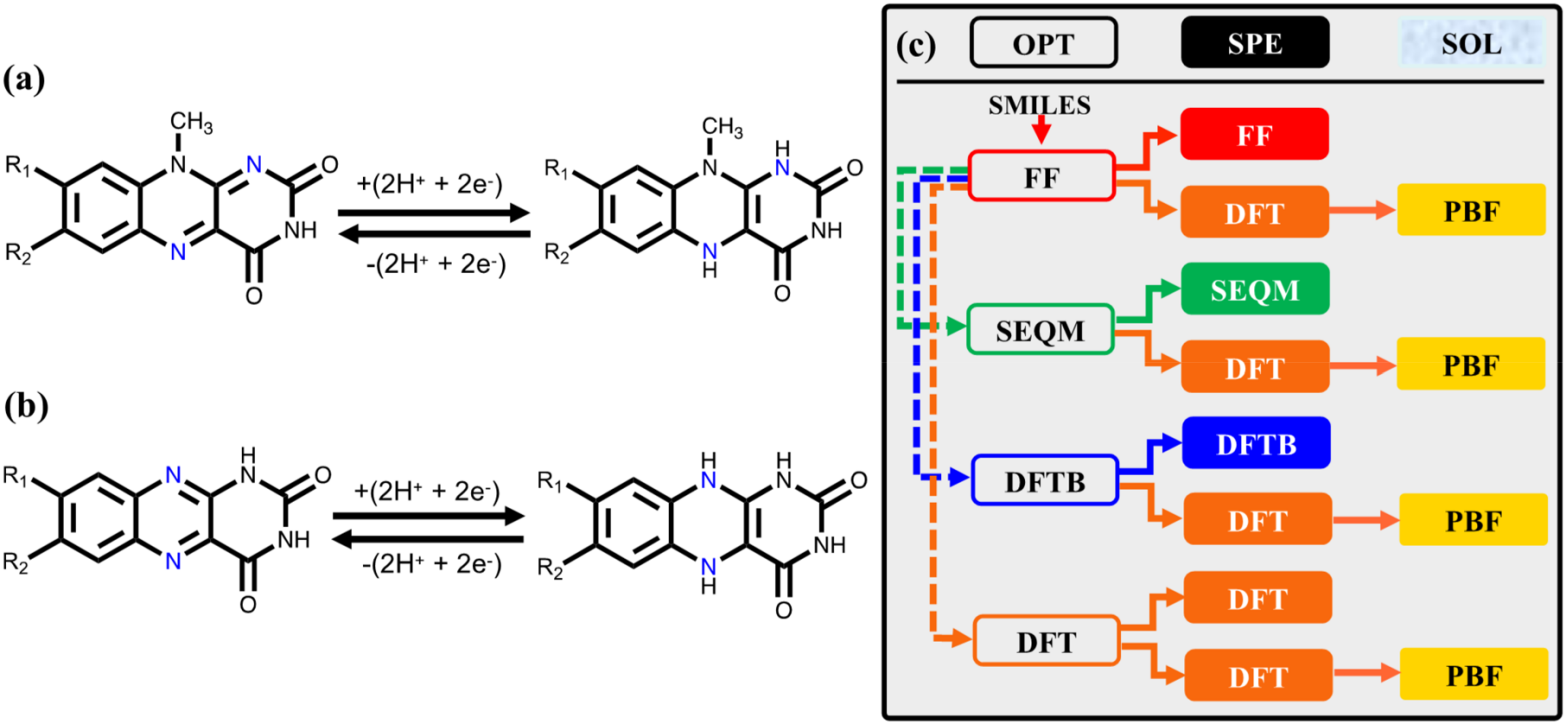
The two-electron–two-proton reaction takes place on (**a**) the heterocyclic nitrogen atoms of the adjacent rings, and (**b**) the heterocyclic nitrogen atoms of the same ring. (**c**) A graphical summary of the various levels of approximations used for estimating $\Delta{E}_{\text{rxn}}$, ${E}_{\text{LUMO}}$ and ${E}_{\text{HOMO}}$ in this work. The text boxes with no background fill color represent geometry optimizations; the boxes with solid color background represent SPE calculations; the boxes with yellow background represent solution phase SPE calculations using an implicit aqueous solvent model. 2D molecule representations have been created using ChemDraw Professional [version 18.0.0.231(4318)] https://www.perkinelmer.com/product/chemdraw-professional-chemdrawpro. Figure has been created using Microsoft PowerPoint [Version 16.16.27 (201012)] https://www.microsoft.com/en-gb/microsoft-365/microsoft-office?rtc=1.

ARFBs are one of the most attractive candidates for grid-scale energy storage due to the independent scaling of their power and energy density^1–3^. The electrolyte, which contains the electroactive compounds for reversible energy storage, is the central component of an ARFB that influences all metrics of battery performance from energy density to rechargeability. One of the most popular electrolyte materials for ARFBs is vanadium^4–6^. However, the economic and technical challenges related to its abundance^7^, high-cost^8,9^ and sluggish reaction kinetics^10,11^ prevent a widespread commercial adoption of the technology. To overcome these limitations, organic electroactive compounds, including quinones^12–14^, viologens^15,16^, TEMPO (2,2,6,6-tetramethyl-1-piperidinyloxy)^17,18^ and their derivatives, have been investigated as electroactive materials in ARFBs. Aza-aromatics, which contain nitrogen atoms in the aryl rings, have also recently been explored as candidate materials^19–24^. In particular, Aziz et al.^19^ and Kwon et al.^23^ have independently reported alloxazines (also called flavins) that show reversible, radical-free redox cycling in alkaline ARFBs with very high current efficiency (99.7%) and capacity retention (> 99.98%) per cycle, as shown in Fig. 1a,b. The battery-relevant physicochemical properties of these molecules can further be improved, for instance, by functionalization with –COOH and/or –OH groups to comply with the practical requirements of high aqueous solubility for ARFBs. HTCS, particularly when powered by quantum chemical calculations, is a promising strategy^25^ for creating virtual libraries of chemically diverse motifs, predicting their performance with descriptors, and for subsequently identifying the most promising candidates for further in-depth studies^19,26–28^. The computational costs associated with the screening of possibly millions of candidate compounds by means of quantum chemical simulations, however, are quite large. Therefore, the performance descriptors for candidate compounds that will be used in a HTCS study need to be chosen carefully, and the trade-offs between the accuracy of descriptors and the computational costs for computing these need to be quantified. One of the central properties of research interest is the redox potential between the redox couples. To date, HTCS studies on various classes of organic compounds have used density functional theory (DFT) calculated reaction energies^19^ and LUMO energies^26–28^ as the default descriptors for predicting redox potentials. Although DFT is a widely accepted method for performing such calculations, there are other computational methods, such as semi-empirical quantum mechanics (SEQM)^29–31^ and density functional tight-binding (DFTB)^32^, that are computationally more affordable, and therefore, worth exploring from the standpoints of accuracy and computing efficiency.

In our recent work on prediction of redox potentials for quinone molecules^33^, we systematically evaluated different computational methods and showed that molecular geometry optimization (OPT) with a low-level computational method followed by DFT calculation of the single point energy (SPE) with implicit aqueous solvation offers an equipollent accuracy as the high-level DFT methods, albeit at significantly (~ 10^3^) lower computational costs^33^. To the best of our knowledge, an analysis of the effect of various factors on the accuracy for predicting redox potentials for alloxazines, such as the level of theory for OPT, the level of theory for the calculation of SPE, and also together with an inclusion/exclusion of solvation effects, has not yet been performed. Furthermore, it is worth exploring the relative performance of various chemical descriptors for predicting the redox potentials of electroactive compounds in general.

To understand how the aforementioned factors affect the prediction accuracies for alloxazines, the performance of various computational methods corresponding to different levels of theoretical fidelity, such as force field (FF)^34,35^ based molecular mechanics, SEQM, DFTB, and DFT, are systematically evaluated in this work. Apart from reaction energies, other energy-based descriptors, such as highest occupied molecular orbital (HOMO) and LUMO are independently calibrated against the measured redox potentials to evaluate their performances. An optimum combination of methods for an accelerated and robust prediction of alloxazine redox potentials is suggested. ﻿The results provide insights on the influential factors that affect the efficiency of computational methods in predicting the redox potentials, which are often overlooked.

## Computational workflow

To make generalizable and consistent comparisons between various computational approaches, we developed a systematic workflow (Fig. 1c). In this workflow, the starting point for any given molecule is its SMILES representation^36^, which is a widely used form of graph-representation. The SMILES representation is at first converted to a two-dimensional (2D) geometrical representation using a SMILES interpreter. Next,

1. The 2D representation is converted to a three-dimensional (3D) geometry by performing OPT with the OPLS3e FF and identifying the lowest energy 3D conformer^34,35^. It is important to note that the FF level geometry is the starting point for all considered theoretical approaches here.
2. Next, gas phase OPT is performed on the 3D geometry at three different levels of theory, namely: SEQM, DFTB, and DFT. OPT is also carried out separately in the implicit aqueous phase but these are not shown in Fig. 1c for the sake of simplicity. This step also yields the corresponding SPEs of molecules that have been calculated at each level of theory.
3. SPEs of the 3D gas-phase geometries from low-level methods are calculated using various DFT functionals. This step yields energy values that are directly comparable but are obtained using geometries that result from four different levels of theory.
4. Finally, for the geometries obtained in Step (2), the SPEs are recalculated, this time by including the effect of an implicit aqueous medium (SOL) using the Poisson–Boltzmann solvation model (PBF)^37,38^.

## Results and discussions

### Comparison of chemical descriptors from DFT

DFT is the highest level of theory considered in this work. Therefore, the performance of the exchange–correlation functionals are discussed first with an aim to use them as benchmarks for the low-level methods. We first compare the performance of total internal energy, $\Delta{U}_{\text{rxn}}$, and Gibbs free energy, $\Delta{G}_{\text{rxn}}^{\text{o}}$, as descriptors for predicting the redox potentials. For this purpose, DFT calculations employing the PBE functional were performed for OPT in the gas-phase and then calculating the SPE in the implicit aqueous-phase. The calibration performances of $\Delta{U}_{\text{rxn}}$ (R^2^ = 0.926, RMSE = 0.021 V) and $\Delta{G}_{\text{rxn}}^{\text{o}}$ (R^2^ = 0.919, RMSE = 0.022 V) are very similar, as shown in Supplementary Fig. S1. The inclusion of zero-point energy (ZPE) in $\Delta{U}_{\text{rxn}}$, as well as entropic effects, in $\Delta {G}_{\text{rxn}}^{\text{o}}$, is not better than using only the reaction energy $\Delta{E}_{\text{rxn}}$ (R^2^ = 0.959, RMSE = 0.016 V). Moreover, the inclusion of these effects is detrimental from an HTCS perspective, not only because of their lower accuracy but also their high computational costs. Therefore, all the following discussions in this work consider only $\Delta{E}_{\text{rxn}}$ as the total energy-related descriptor, besides the orbital energy-related descriptors, i.e., the LUMO energy (${E}_{\text{LUMO}}$) and the HOMO energy (${E}_{\text{HOMO}}$).

**Figure 2:**
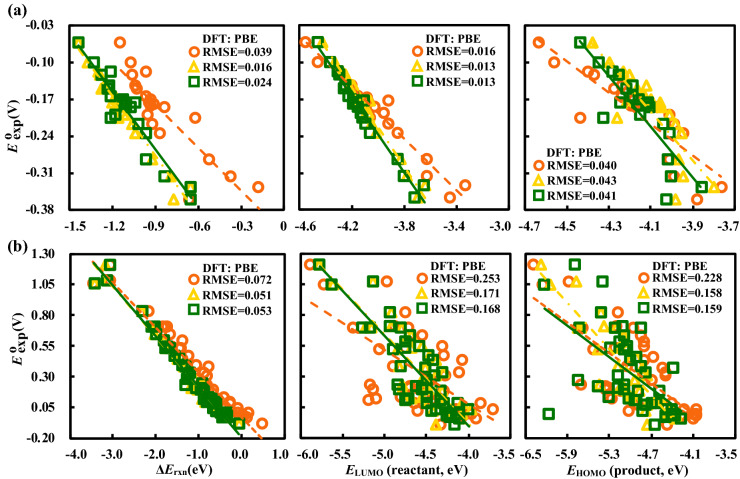
Scatter plots showing linear correlations of the PBE calculated $\Delta{E}_{\text{rxn}}$, ${E}_{\text{LUMO}}$, and ${E}_{\text{HOMO}}$, versus the experimentally measured redox potentials (${E}_{\text{exp}}^{\text{o}}$) for (**a**) alloxazine-based compounds, and (**b**) quinone-based compounds. The color orange represents both the OPT and SPE in gas-phase, the color yellow represents OPT in gas-phase followed by SPE with SOL, and the color green represents both OPT and SPE with SOL.

Next, according to the three computational schemes discussed in the Computational workflow, a comparison of $\Delta{E}_{\text{rxn}}$, ${E}_{\text{LUMO}}$*,* and ${E}_{\text{HOMO}}$, at the PBE level is shown in Fig. 2a for the alloxazine compounds. Clearly, the reactant’s ${E}_{\text{LUMO}}$ (R^2^ = 0.974, RMSE = 0.013 V) emerges as the best descriptor, which is followed closely by $\Delta{E}_{\text{rxn}}$ (R^2^ = 0.959, RMSE = 0.016 V) and then the product’s $\Delta{E}_{\text{HOMO}}$ (R^2^ = 0.743, RMSE = 0.040 V), irrespective of whether the OPT and SPE calculations are performed in the gas-phase (orange markers) or in the aqueous-phase (green markers). This ranking of descriptors was found to be consistent for all the 11 exchange–correlation functionals considered in this work (see Supplementary Fig. S2). These results imply that for HTCS on alloxazines, the computational effort can be reduced at least by half simply by using ${E}_{\text{LUMO}}$ as a descriptor for the experimental redox potential. Moreover, a large variety of DFT methods are not able to capture either the energetics or the geometry of the reduced forms of the alloxazines to a comparable level of accuracy as the oxidized forms of these compounds. Furthermore, the ranking of the descriptors considered here are in stark contrast to the descriptors for quinones (Fig. 2b)^33^. For quinones, $\Delta{E}_{\text{rxn}}$ (R^2^ = 0.977, RMSE = 0.051 V) is clearly the best descriptor across all the three computational schemes, followed by ${E}_{\text{HOMO}}$ (R^2^ = 0.779, RMSE = 0.158 V) and ${E}_{\text{LUMO}}$ (R^2^ = 0.748, RMSE = 0.168 V). The difference in performance of the descriptors for alloxazines and quinones reveal that such comparisons of methods and descriptors are needed for other classes of organic electroactive compounds.

**Figure 3:**
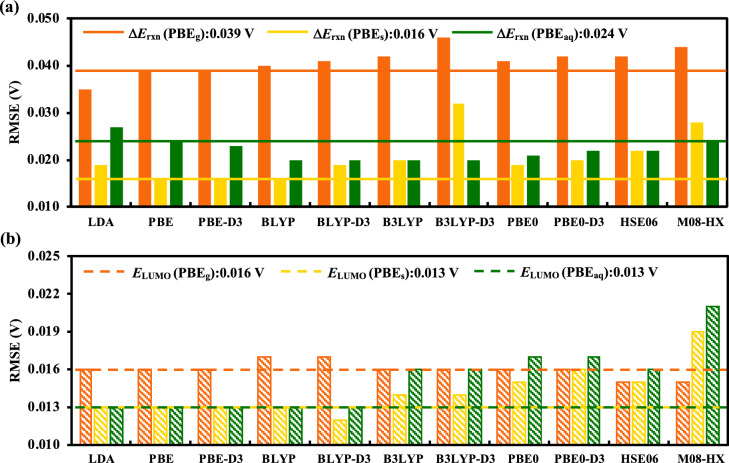
Performance comparison of various exchange–correlation functionals for predicting ${E}_{\text{exp}}^{\text{o}}$. The bar plots (**a**) and (**b**) show the RMSEs for descriptors $\Delta{E}_{\text{rxn}}$ and ${E}_{\text{LUMO}}$, respectively. The color orange represents both OPT and SPE in gas-phase, the color yellow represents OPT in gas-phase followed by SPE with SOL, and the color green represents both OPT and SPE with SOL.

Another key aspect of comparisons is the computational scheme used for the calculations of the descriptors. For these comparisons, we ignore ${E}_{\text{HOMO}}$ descriptor, since it performs significantly worse than both ${E}_{\text{LUMO}}$ and $\Delta{E}_{\text{rxn}}$ for all the quantum chemical methods (see Supplementary Fig. S2 and Table S2). Three kinds of schemes for computing the descriptors have been devised using each of the 11 exchange–correlation functionals, as follows: (A) with gas-phase OPT and SPE calculation, (B) with OPT in the gas-phase and the following SPE calculation in an implicit aqueous environment (SOL), and (C) with both the OPT and SPE in SOL. The performance of the various exchange–correlation functionals are compared using bar plots of RMSE and R^2^ values, as shown in Fig. 3 and Supplementary Fig. S3, respectively. In Fig. 3, the subscript ‘g’ corresponds to scheme (A), ‘s’ corresponds to scheme (B), and ‘aq’ corresponds to scheme (C). When compared under the same set of approximations, it is observed that:

I. When using $\Delta{E}_{\text{rxn}}$ in scheme (A), LDA_g_ (R^2^ = 0.801, RMSE = 0.035 V) and followed closely by PBE_g_ (R^2^ = 0.756, RMSE = 0.039 V) are the two best performing methods. Inclusion of higher order exchange effects and parametrizations, such as in HSE06 and M08-HX, are found to have no positive effect on the prediction accuracies, which is in clear contrast to the case of quinones that have been reported earlier^33^. With $\Delta{E}_{\text{rxn}}$ in scheme (B) (i.e., a hybrid scheme), PBE_s_ (R^2^ = 0.959, RMSE = 0.016 V) emerges as the best performing method and shows a significant improvement over the gas-phase only scheme. A decrease in RMSE upon inclusion of SOL is observed across all the 11 functionals, but to varying degrees. Finally, when using $\Delta{E}_{\text{rxn}}$ in scheme (C), BLYP-D3_aq_ (R^2^ = 0.937, RMSE = 0.020 V) is the best performing method that is followed very closely by BLYP_aq_ (R^2^ = 0.935, RMSE = 0.020 V). Inclusion of SOL during OPT was found to worsen the prediction accuracies with respect to the hybrid scheme in all cases except for calculations employing B3LYP-D3 and M08-HX. Given the fact this scheme is also computationally much more demanding, there is no advantage of using it any further. These findings are in accordance with those of the quinone molecules^33^. An overall conclusion is that at the GGA-DFT level (PBE, BLYP), it is possible to use $\Delta{E}_{\text{rxn}}$ as a descriptor to predict ${E}_{\text{exp}}^{\text{o}}$ for alloxazines within a range of common experimental errors (i.e. ~ 50 mV).
II. When using ${E}_{\text{LUMO}}$ in scheme (A), almost all methods show very similar performances. Inclusion of higher order exchange effects and parametrizations in the form of DFT functionals is found to have a small positive effect on the prediction accuracy. With ${E}_{\text{LUMO}}$ in the hybrid scheme (B), most methods show similar and improved performance, with BLYP-D3_s_ (R^2^ = 0.976, RMSE = 0.012 V) emerging as the best performing method that is followed closely by PBE_s_ (R^2^ = 0.974, RMSE = 0.013 V). A decrease in RMSE upon inclusion of SOL is observed across all DFT methods, except for M08-HX. Finally, when using ${E}_{\text{LUMO}}$ in scheme (C), BLYP-D3_aq_ (R^2^ = 0.974, RMSE = 0.013 V) is again the best performing method followed closely by PBE_aq_ (R^2^ = 0.972, RMSE = 0.013 V). Inclusion of SOL during OPT was found to worsen the prediction accuracy with respect to the hybrid scheme for the hybrid functionals such as B3LYP, HSE06, and M08-HX. Yet again, there is no advantage of using this scheme as it is computationally much more demanding. An overall conclusion is that at the GGA-DFT level, it is possible to use ${E}_{\text{LUMO}}$ as a descriptor to predict ${E}_{\text{exp}}^{\text{o}}$ for alloxazines within the range of common experimental errors.

#### Discussion

Widely used GGA-level DFT methods are good enough for predicting the redox potentials of alloxazines within the range of common experimental errors, irrespective of whether $\Delta{E}_{\text{rxn}}$ or ${E}_{\text{LUMO}}$ are chosen as the descriptors. Additionally, there is a positive effect on the prediction accuracy due to the inclusion of implicit solvation during the calculations of $\Delta{E}_{\text{rxn}}$ and ${E}_{\text{LUMO}}$. The positive effect is also observed when using ${E}_{\text{LUMO}}$ of the reactant as a descriptor, which indicates that the interactions of the reactant’s aromatic rings and H-bonds with the surrounding medium are quite influential, as was also observed in the case of quinone-based molecules^33^. The relative performance of the two descriptors doesn’t change when MAE is used as the metric, as can be seen at the end of Tables S1a,b. Upon using the benchmark PBE functional, it is found that molecules with serial no: 15, 19, and 20, have considerably higher prediction errors than others for both descriptors.

From a theoretical point of view, optimization of geometries and calculation of energies in a solvated environment should ideally yield the best answer. However, it is observed that optimizing geometry using the PBF implicit solvation model worsens prediction accuracies, even if by a very small amount, across all the DFT flavors (LDA, GGA, Hybrid and meta-GGA). Without loss of generality, it can be argued that there can be two main sources of errors when using implicit solvation models, namely, erroneous geometry optimization and/or erroneous energy estimation. In order to determine possible sources of contributions to the overall error, we performed additional simulations in which the geometry was optimized with the PBF solvation model, however, the energy was then calculated in the gas phase. From a modelling perspective, these simulations do not correspond to a meaningful approximation of the “real” physics. However, it is very revealing to notice that the errors under these approximations are much worse (R^2^ = 0.681, RMSE = 0.043 V) than the fully gas phase treatment of the molecules (R^2^ = 0.756, RMSE = 0.039 V) with $\Delta{E}_{\text{rxn}}$ as the descriptor, as seen in Fig. S5a. The same observation holds true when using ${E}_{\text{LUMO}}$ as the descriptor although the results are only slightly worse for the aforementioned scheme (R^2^ = 0.953, RMSE = 0.017 V) with respect to the fully gas phase treatment (R^2^ = 0.956, RMSE = 0.016 V), as seen in Fig. S5b. As the energy is calculated in gas phase under both approximations, the error most likely originates in the geometry. Based on these observations, it can be argued that the PBF solvation model is not accurate enough to improve the gas phase geometry, which is the likely source of error. It is also possible that the estimation of geometry is more erroneous for product molecules than the reactant molecules because the increase in error when using ${E}_{\text{LUMO}}$ is not as significant as the case of $\Delta{E}_{\text{rxn}}$.

To investigate the second aspect of the dependence of energy on the implicit solvation model itself, we first note that given the availability of several other solvation models in literature, it is possible that they will produce different results. As an example, in the work of Kim et al.^39^, it was shown that the prediction of reduction potentials of Anthraquinones in aqueous solutions is prone to errors due to overestimation of the intramolecular H-bond interactions when using the PCM (Bondi) implicit solvation model. Further, they showed that QM/MM calculations, with the TIP3P force field used explicitly for the water molecules, alleviate the overestimation and lead to a more balanced treatment of solute–solvent interactions. To the best of our knowledge, there are no known studies in literature that use high fidelity methods such as QM/MM for prediction of redox potentials of alloxazine molecules. Performing QM/MM simulations is not yet suitable from an HTCS perspective and is out of the scope of this study. Therefore, it cannot be confirmed if intramolecular H-bond or other interactions also influence the accuracy of implicit solvation models for treatment of alloxazines. Nevertheless, we performed additional simulations on alloxazines with the PCM (COSMO) model, which is widely used for aqueous systems. As can be seen in Fig. S5c,d, the overall conclusions remain the same and the performance of the PCM (COSMO) is strikingly similar to that of the PBF model used in this study, under every approximation and for both of the descriptors. We believe that systematic improvements in characterization of solvation effect likely need to go beyond implicit models. Lastly, there might be a serendipitous cancellation of errors when using the gas-phase geometry that is affected by the changes in the geometry due to the implicit solvation model in use. However, this claim is not possible to verify given the relatively small calibration set and scope of methods covered in this study.

Nevertheless, these results are important from the standpoint of computational efficiency, because even when starting from a DFT computed gas-phase geometry, performing a geometry optimization with implicit solvation is computationally about twice more demanding than without it. For the study of alloxazine-based compounds, we propose a hybrid scheme of using gas-phase geometries and then performing DFT SPE calculations on them to improve the prediction accuracies. The various DFT functionals, in this work, were compared solely on the basis of their performance in predicting the measured potentials. Exchange–correlation functionals that contain high degrees of empiricism, such as M08-HX^40^, are aimed at producing improved values for a chosen set of physically observable properties. However, such heavily parametrized functionals tend to produce less accurate electron densities than the ones with little to no empiricism in their designs, such as PBE functional^41^. Accordingly, we chose PBE as the benchmark DFT functional among all the compared DFT functionals, as it offers the best compromise between the accuracy in results and the cost of calculations. Notably, the PBE functional was also found to show very good performance for quinone-based molecules in our recent work^33^. Accordingly, for the prediction of redox potentials versus RHE at pH = 7, the equation employing the DFT(PBE) calculated ${E}_{\text{LUMO}}$ of the reactant alloxazine compound is:

1 $${E}^{o}=-0.376[{E}_{\text{LUMO}}]-1.726$$

### Comparison of chemical descriptors from low-level methods: FF, SEQM, and DFTB

After establishing the effectiveness of quantum chemical methods as a benchmark, we analyze the computationally less costly low-level methods for optimizing geometries and predicting the energies of molecules. As summarized in Fig. 1, various low-level methods, including FF, SEQM, and DFTB, have been employed for calculations. Here again, ${E}_{\text{HOMO}}$ is ignored as a descriptor because it performs worse than both $\Delta{E}_{\text{rxn}}$ and ${E}_{\text{LUMO}}$ for all the low-level methods. The descriptors are calculated using the following three schemes:

I. $\Delta{E}_{\text{rxn}}$ and ${E}_{\text{LUMO}}$ are taken directly from the results of low-level method geometry optimizations either in gas- or aqueous-phases, with the exception of LUMO energies that are not available from FF calculations.
II. $\Delta{E}_{\text{rxn}}$ and ${E}_{\text{LUMO}}$ values are taken from gas-phase DFT calculations employing the PBE functional on the molecular geometries obtained through scheme (I). Descriptor values in this scheme are henceforth abbreviated as DFT-SPE_g_.
III. $\Delta{E}_{\text{rxn}}$ and ${E}_{\text{LUMO}}$ values are taken from DFT calculations employing the PBE functional with implicit solvation on the molecular geometries obtained through scheme (I). Descriptor values in this scheme are henceforth abbreviated as DFT-SPE_s_.

**Figure 4:**
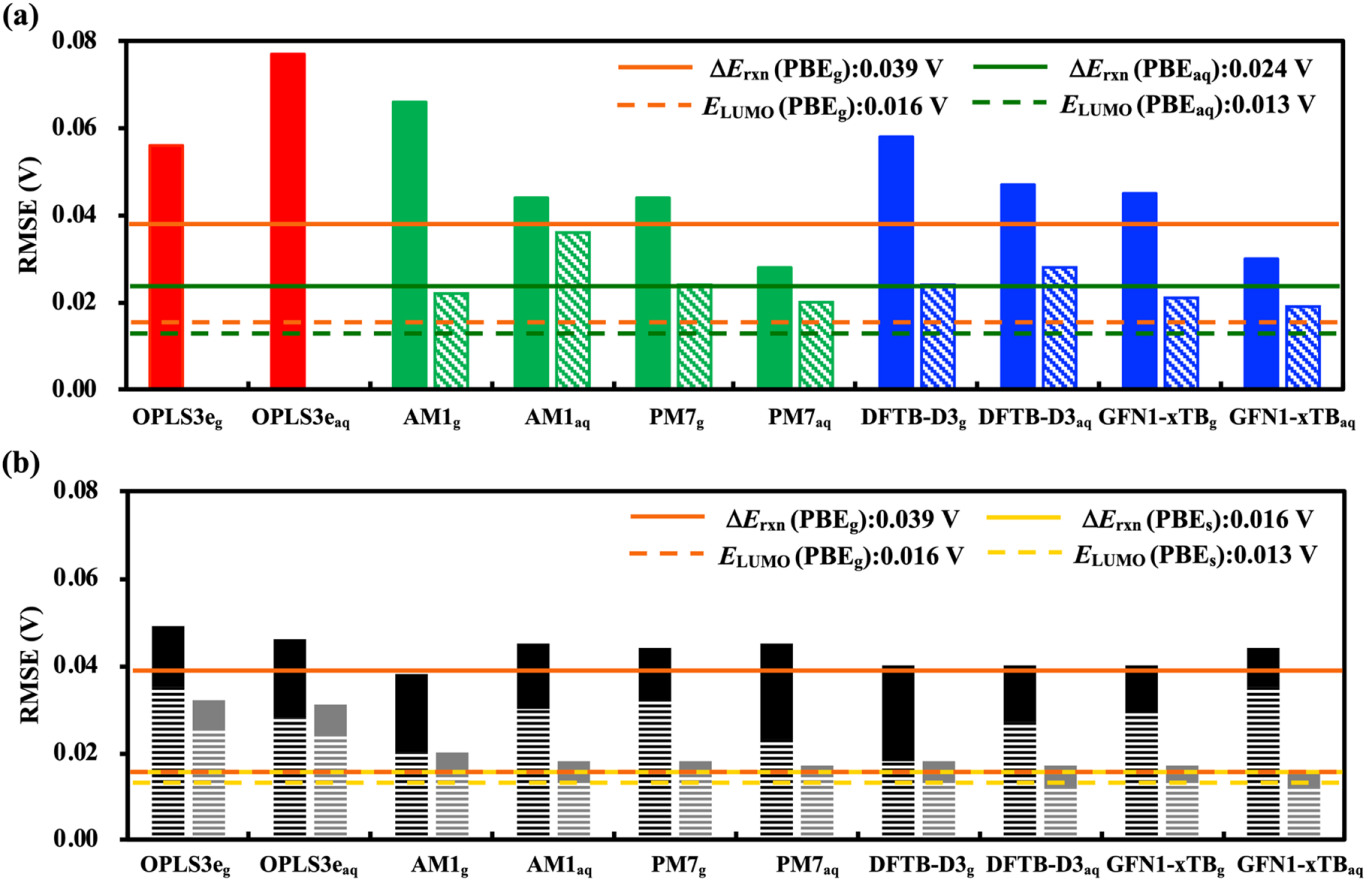
Performance comparison of low-level methods (FF, SEQM, and DFTB) considered in the current work. (**a**) RMSEs of the descriptors computed at the three low-level methods. (**b**) RMSEs for the DFT (PBE) SPE calculations on the geometries obtained from the three low-level methods. In (**a**) the solid bars show RMSEs for the descriptor $\Delta{E}_{\text{rxn}}$ and the hashed bars show the RMSEs for the descriptor ${E}_{\text{LUMO}}$. In (**b**) the solid bars show results using SPE without implicit solvation and the hashed bars show the results with it. The horizontal lines represent the benchmarks established in Fig. 3.

From Fig. 4 and Supplementary Fig. S4, several observations are made on comparing the RMSE and R^2^ data across the various method combinations when using either of $\Delta{E}_{\text{rxn}}$ and ${E}_{\text{LUMO}}$ as the descriptor. To simplify, we only discuss the best method from each low-level calculation category. The detailed performance metrics of all methods and their variations considered in the current work have been provided in Supplementary Tables S3–S7.

#### Comparisons within scheme (I) using $\Delta{{{E}}}_{\text{r}\text{x}\text{n}}$

when comparing predictions using $\Delta{E}_{\text{rxn}}$ to ${\text{PBE}}_{\text{g}}$ (R^2^ = 0.756, RMSE = 0.039 V) and to ${\text{PBE}}_{\text{aq}}$ (R^2^ = 0.910, RMSE = 0.024 V) benchmarks, it is observed in Fig. 4a (solid green bar) that the best performing SEQM method ${\text{PM}7}_{\text{g}}$ shows significantly better performance compared to the FF method. Aqueous-phase geometry optimization with the COSMO solvation model leads to better prediction accuracy for ${\text{PM}7}_{\text{aq}}$, however, both the gas- and aqueous-phase performances are still below than their corresponding PBE benchmarks. The best performing DFTB method GFN1-xTB_g_ shows a very similar performance to ${\text{PM}7}_{\text{g}}$. The aqueous-phase geometry optimization with the COSMO solvation model leads to better prediction accuracy for GFN1-xTB_aq_.

#### Comparisons within scheme (I) using $\Delta{{{E}}}_{\text{L}\text{U}\text{M}\text{O}}$

When comparing predictions using reactant ${E}_{\text{LUMO}}$ to ${\text{PBE}}_{\text{g}}$ (R^2^ = 0.956, RMSE = 0.016 V) and to ${\text{PBE}}_{\text{aq}}$ (R^2^ = 0.972, RMSE = 0.013 V) benchmarks, it is observed in Fig. 4a (hashed green bar) that the gas-phase ${\text{AM}1}_{\text{g}}$ and ${\text{PM}7}_{\text{g}}$ methods show good prediction accuracies, but they are still much worse than the ${\text{PBE}}_{\text{g}}$ benchmark. Aqueous-phase ${\text{PM}7}_{\text{aq}}$ geometry optimizations with the COSMO solvation model improves prediction accuracy. On the contrary, the aqueous-phase ${\text{AM}1}_{\text{aq}}$ optimization leads to a worse performance. The best performing gas-phase DFTB method GFN1-xTB_g_ shows similar results to ${\text{AM}1}_{\text{g}}$. Aqueous-phase GFN1-xTB_aq_ optimization shows a slightly improved performance.

#### Comparisons within scheme (II) using $\Delta{{{E}}}_{\text{r}\text{x}\text{n}}$

When comparing predictions using $\Delta{E}_{\text{rxn}}$, it is observed in Fig. 4b (solid black bars) that DFT-SPE_g_ computations on the gas-phase SEQM geometries show improved prediction accuracies with respect to their counterparts from scheme (I). The best performing method is ${\text{AM}1}_{\text{g}}$, with its prediction accuracy being surprisingly better than the ${\text{PBE}}_{\text{g}}$ benchmark. DFT-SPE_g_ calculations on ${\text{AM}1}_{\text{aq}}$ optimized geometries lead to worse predictions when compared to its counterpart from scheme (I). DFT-SPE_g_ on DFTB-D3_g_ optimized geometries reach a performance close to the ${\text{PBE}}_{\text{g}}$ benchmark. DFT-SPE_g_ calculations on aqueous-phase DFTB-D3_aq_ geometries resulted in worse predictions, as was also observed for the SEQM methods.

#### Comparisons within scheme (II) using ${E}_{\text{L}\text{U}\text{M}\text{O}}$

When comparing predictions using reactant ${E}_{\text{LUMO}}$, it is observed in Fig. 4b (solid grey bars) that DFT-SPE_g_ calculations on the best performing gas-phase ${\text{PM}7}_{\text{g}}$ geometries show significantly improved prediction accuracy with respect to their counterparts from scheme (I), reaching close to the ${\text{PBE}}_{\text{g}}$ benchmark. DFT-SPE_g_ calculations on aqueous-phase ${\text{PM}7}_{\text{aq}}$ geometries also show better performance. DFT-SPE_g_ computations on the best performing gas-phase GFN1-xTB_g_ geometries show slightly improved prediction accuracy when compared to their counterparts from scheme (I), but still reaches close to the ${\text{PBE}}_{\text{g}}$ benchmark. DFT-SPE_g_ computations on aqueous-phase GFN1-xTB_aq_ geometries also result in better predictions.

#### Comparisons within scheme (III) using ${E}_{\text{r}\text{x}\text{n}}$

When comparing predictions using ${E}_{\text{rxn}}$ to the ${\text{PBE}}_{\text{s}}$ (R^2^ = 0.959, RMSE = 0.016 V) benchmark, it is observed in Fig. 4b (hashed black bars) that RMSEs are significantly lower by 0.014 V and 0.018 V for DFT-SPE_s_ calculated on ${\text{OPLS}3\text{e}}_{\text{g}}$ and ${\text{OPLS}3\text{e}}_{\text{aq}}$ geometries, respectively, in comparison to their gas-phase counterparts from scheme (II). The prediction accuracy for DFT-SPE_s_ calculations on the best performing ${\text{AM}1}_{\text{g}}$ and ${\text{AM}1}_{\text{aq}}$ geometries is improved significantly when compared to its counterpart in scheme (II). Similar improvements are observed for DFT-SPE_s_ calculations on the best performing DFTB-D3_g_ and DFTB-D3_aq_ geometries.

#### Comparisons within scheme (III) using ${E}_{\text{L}\text{U}\text{M}\text{O}}$

When comparing predictions using reactant ${E}_{\text{LUMO}}$ to the ${\text{PBE}}_{\text{s}}$ (R^2^ = 0.974, RMSE = 0.013 V) benchmark, it is observed in Fig. 4b (hashed grey bars) that the performance of DFT-SPE_s_ calculations on geometries from all lower level methods show improvements than their counterparts from scheme (II), however, these improvements are not as pronounced as those for $\Delta{E}_{\text{rxn}}$.

#### Discussion

Several conclusions can be derived after comparing the performance metrics of low-level computational methods, including on the basis of DFT(PBE) calculation of SPE on the frozen coordinates, both with and without implicit solvation effects. First, similar to the case of DFT methods, the reactant’s ${E}_{\text{LUMO}}$ is a better descriptor than the $\Delta{E}_{\text{rxn}}$ of redox reaction for both the SEQM and DFTB methods. Secondly, gas-phase PBE calculations of SPE on the frozen coordinates show improved prediction accuracies for all the low-level methods. This observation implies that the computationally costly DFT geometry optimizations of the reactant molecules are hardly necessary, especially for a first-order screening of a large number of candidate compounds. Thirdly, like the case of DFT methods, the computational effort can approximately be halved when the reactant ${E}_{\text{LUMO}}$ is used for the prediction of potentials, particularly by using either of the SEQM (PM7, AM1) or DFTB (DFTB-D3, GFN1-xTB) methods. Lastly, irrespective of the chosen descriptor, for all low-level methods, the inclusion of implicit aqueous solvation during the DFT calculation of SPE on the gas-phase geometries leads to an improved prediction accuracy that reaches to within 20 meV of the DFT benchmark. From the findings made in this study, we recommend SEQM and DFTB as practical methods based on the trade-offs between computational costs and prediction accuracies. Accordingly, the equation to predict redox potentials versus RHE at pH = 7, by employing the DFTB(GFN1-xTB_aq_) calculated ${E}_{\text{LUMO}}$ of the reactant alloxazine compound is:

2 $${E}^{\text{o}}=-0.373[{E}_{\text{LUMO}}]-3.825$$

### Effects of implicit solvation on the prediction performance

**Figure 5:**
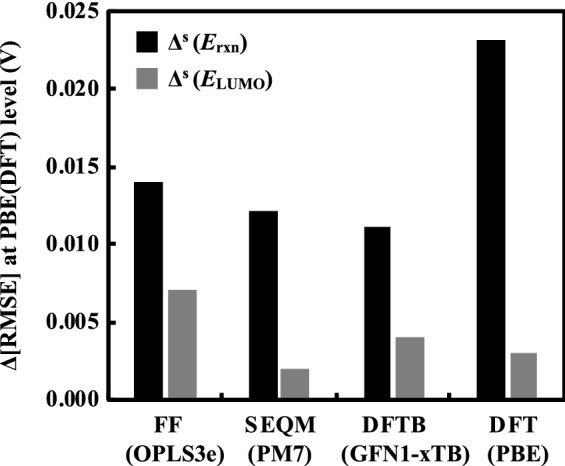
Bar plot showing the decrease in error, Δ[RMSE], due to the inclusion of implicit solvation. A larger value of Δ^s^ implies higher improvement in the accuracy of the predicted results. The black solid bars represent Δ^s^ (${E}_{\text{rxn}}$), whereas the grey solid bars represent Δ^s^ (${E}_{\text{LUMO}}$).

Figure 5 shows the decrease in error, Δ[RMSE], as a result of including implicit solvation during DFT calculation of SPE (Δ^s^) for the representative methods from four different methodological levels considered in this work. As shown with solid black bars, when $\Delta{E}_{\text{rxn}}$ is used as the descriptor, Δ^s^ (in V) are 0.014 (OPLS3e), 0.012 (PM7), 0.011 (GFN1-xTB), and 0.023 (PBE). When ${E}_{\text{LUMO}}$ is used as the descriptor, as shown with solid grey bars, Δ^s^ are 0.007 (OPLS3e), 0.002 (PM7), 0.004 (GFN1-xTB), and 0.003 (PBE). Clearly for ${E}_{\text{LUMO}}$, Δ^s^ is smaller at each level of theory when compared to the case of $\Delta{E}_{\text{rxn}}$. We postulate that the reason for this difference is the presence of additional H-bonds in the products, due to which the solvation effects become more pronounced. These findings are also expected to be useful for improving the cheminformatics and advanced machine learning models that employ a descriptor-based approach to predict the solubility of compounds in water^42^.

## Methods

### Thermodynamic principle

The thermodynamic basis to predict the redox potentials of electroactive alloxazine compounds for ARFBs is the aqueous-phase redox reaction given by Eq. (3):

3 $$\text{Z}+{2\text{H}}^{+}{+ 2e}^{- }\to {\text{ZH}}_{2}$$

This redox reaction assumes a rapid and reversible two-electron two-proton mechanism in which the product, ZH_2_, is generated from the reactant, Z. In this work, the calculated reaction energy, $\Delta{E}_{\text{rxn}}=E\left({\text{ZH}}_{2}\right)-\left[E\left(\text{Z}\right)+E\left({\text{H}}_{2}\right)\right]$, is used as a descriptor for predicting the redox potential, under the same set of assumptions as described in our recent work on quinones^33^. In principle, the reaction Gibbs free energy, $\Delta{G}_{\text{rxn}}^{\text{o}}$, is related to the redox potential, ${E}^{\text{o}}$, through the Nernst equation given by ${E}^{\text{o}}=-\Delta{G}_{\text{rxn}}^{\text{o}}/nF$. However, as discussed above, neither the $\Delta{G}_{\text{rxn}}^{\text{o}}$ nor the internal energy $\Delta{U}_{\text{rxn}}$ that includes the zero-point energy corrections to $\Delta{E}_{\text{rxn}}$, are found to offer improved prediction accuracies in comparison to the $\Delta{E}_{\text{rxn}}$. Apart from $\Delta{E}_{\text{rxn}}$, the energy corresponding to the LUMO, ${E}_{\text{LUMO}}$, of the reactant molecule $\text{Z}$ is also considered as a key descriptor because the reduction of $\text{Z}$ implies filling of its LUMO, and because the location of ${E}_{\text{LUMO}}$ with respect to the electrode Fermi level indicates the thermodynamic driving for electron transfer. Using similar arguments, the energy level corresponding to the HOMO, ${E}_{\text{HOMO}}$, of the product molecule ${\text{ZH}}_{2}$ is a key descriptor because the oxidation of ${\text{ZH}}_{2}$ implies emptying of its HOMO. As explained below, we used various computational chemistry methods for the calculation of $\Delta{E}_{\text{rxn}}$, ${E}_{\text{LUMO}}$ and ${E}_{\text{HOMO}}$, and evaluated their performances in predicting the experimentally measured redox potentials.

### Computational details

In this work, the MacroModel program is used for the FF^34,35^ based configurational searches and OPT, and the Jaguar program^38^ is employed for DFT calculations, all as implemented in the Schrödinger Materials Science Suite (version 2019-2). The SEQM (MOPAC) and DFTB calculations are performed using the ADF program^43^. The molecular structures of redox couples are optimized both in the gas- and aqueous-phases using the OPLS3e FF that provides a broad coverage of small compounds^34,35^. The aqueous-phase geometry optimizations at FF level use a generalized Born model implemented in the Schrödinger’s MacroModel program. In addition, a FF based exhaustive conformational search over rotatable bonds and torsional interactions is performed using the MacroModel program to determine the lowest energy conformers for each molecule. These lowest energy conformers are then used as inputs to perform the gas- and aqueous-phase geometry optimizations using nine different SEQM methods, including AM1^44^, MNDO^45^, MNDOD^46^, PM3^47^, PM6^48^, PM6-D3^49^, PM6-D3H4X^29^, PM7^50^ and RM1^51^. The gas-phase FF optimized geometries are also used as inputs for DFTB level optimizations using the DFTB-D3^52^ and GFN1-xTB^53,54^ methods. The DFTB-D3 computations are performed with a self-consistent charge cycle using the QuasiNANO-2015^32^ parameter set, while the parameters for GFN1-xTB are taken from the work of Grimme et al.^53,54^. The aqueous-phase geometry optimizations at the SEQM and DFTB levels are performed using the COSMO-RS solvation model^55–57^. The choice of this solvation method is constrained by the current availability in the ADF program. Finally, FF minimized geometries are used as inputs to perform geometry optimizations in the gas-phase at the DFT level by using local density approximation (LDA)^58^, generalized gradient approximation (GGA), hybrid, and meta-GGA functionals, which lie on four different rungs of the so-called Jacob’s ladder of accuracy^59^, and vary drastically in their accounting of the exchange–correlation energy. A total of 11 functionals, also including some of the D3 dispersion^60^ corrected variants, are used for OPT and SPE calculations. These functionals include LDA^58^, PBE^41,61^, PBE-D3^60^, BLYP^62^, BLYP-D3^60^, B3LYP^62^, B3LYP-D3^60^, PBE0^63^, PBE0-D3^60^, HSE06^64^, and M08-HX^40^. For the geometries that have been obtained from FF, SEQM, and DFTB optimizations, the DFT level SPEs are computed in the gas-phase, and subsequently in the aqueous-phase using only the PBE functional due to reasons discussed above.

### Calibration data and performance metrics

The experimental redox potential data was collected from a total of 21 alloxazine-based redox couples in neutral and alkaline aqueous solutions. For consistency, all measured redox potentials were corrected to reversible hydrogen electrode (RHE) at pH = 7. In consideration of the generality of calibration models, experimental data on both core molecules as well as their derivatives functionalized with various groups, such as –CH_3_, –Cl, –F, –OMe, –NMe_2_, –CN, COOH, –OCH_3_, –OH and –CH_3_ (see Supplementary Table S1), has been utilized. Accordingly, the calibration data spans a broad range of redox potentials between − 0.359 and − 0.062 V.

It is important to note that the alloxazines synthesized by Rizzo et al.^65^ and Aziz et al.^19^ have different pairs of heterocyclic nitrogen atoms that react. As shown in Fig. 1, in one group of molecules (from Rizzo et al.) the protonation reaction takes place on the heterocyclic nitrogen atoms of the adjacent rings, while in the other (from Aziz et al.) it takes place on the heterocyclic nitrogen atoms of the same ring. In the current work, however, the two types are not treated distinctly because a generic predictive model is sought. The correlations between experiments and calculations are expressed in terms of the commonly used coefficients, namely, the coefficient of determination (R^2^), root-mean-square error (RMSE) and mean absolute error (MAE). R^2^, RMSE, and MAE are calculated using the definitions from the Originlab, in which R^2^, RMSE, and MAE are given by Eqs. (4), (5) and (6), respectively:

4 $$\text{R}^{2} = 1-\frac{\sum_{i=1}^{n}{\left({y}_{i}-{\widehat{y}}_{i}\right)}^{2}}{\sum_{i=1}^{n}{\left({y}_{i}-\stackrel{-}{y} \right)}^{2}}$$

5 $$\text{RMSE }=\sqrt{\frac{{\sum }_{i=1}^{n}{\left({y}_{i}-{\widehat{y}}_{i}\right)}^{2}}{n-1}}$$

6 $$\text{MAE }= \frac{\sum_{i=1}^{n}|{\widehat{y}}_{i}-{y}_{i}|}{n}$$

where ${y}_{i}$ is the experimental measurement made at the ith *x*-value in the data set, ${\widehat{y}}_{i}$ is the predicted response for the measurement, $\stackrel{-}{y}$ is mean of $y$-value. The *x*-value in this study refers to either of $\Delta{E}_{\text{rxn}}$, ${E}_{\text{LUMO}}$ or ${E}_{\text{HOMO}}$, and $y$-value refers to predicted redox potential as described above.

## Supplementary Information

Supplementary Information.

## Data Availability

The generated computational data of compounds is provided in Supplementary Tables S1 to S7.
