# Supplementary material for: A quantitative evaluation of computational methods to accelerate the study of alloxazine-derived electroactive compounds for energy storage
Source: Sci Rep. 2021 Feb 18;11:4089. doi: 10.1038/s41598-021-83605-2 (PMC7892830; doi:10.1038/s41598-021-83605-2)
Supplement: Supplementary file 1 — Supplementary Information. [file 41598_2021_83605_MOESM1_ESM.pdf]

Supplementary Information

A quantitative evaluation of computational methods to accelerate the study of alloxazine-derived electroactive compounds for energy storage

Qi Zhang[1,2,3], Abhishek Khetan[1,2], Süleyman Er[1,2]*

[1] DIFFER – Dutch Institute for Fundamental Energy Research, De Zaale 20, 5612 AJ Eindhoven, The Netherlands

[2] CCER – Center for Computational Energy Research, De Zaale 20, 5612 AJ Eindhoven, The Netherlands

[3] Department of Applied Physics, Eindhoven University of Technology, 5600 MB Eindhoven, The Netherlands

[*] E-mail: s.er@differ.nl

Explanation of the abbreviations used in the below tables:

SMILES: simplified molecular-input line-entry system

FF: force field

SEQM: semi-empirical quantum mechanics

DFTB: density functional tight-binding

DFT: density functional theory

OPT: geometry optimization

SPE: single point energy

SOL: implicit aqueous medium

R^2: coefficient of determination

RMSE: root-mean-square error

MAE: mean absolute error

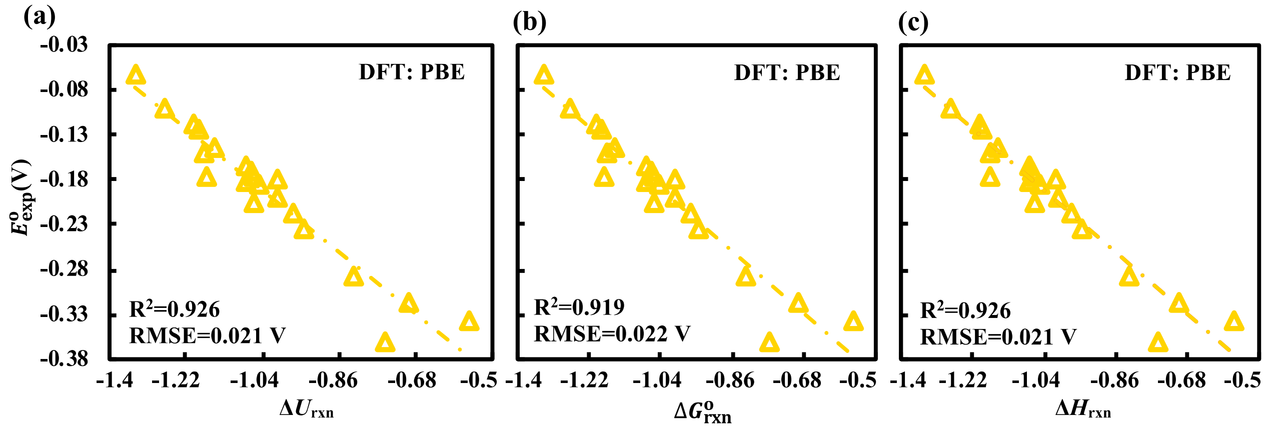

Figure S1. Performance of (a) ΔU_{rxn}, (b) $\Delta G^{\mathrm{o}}_{\mathrm{rxn}}$, and (c) ΔH_{rxn} as descriptors for the prediction of experimentally measured redox potentials, $E^{\mathrm{o}}_{\mathrm{exp}}$. The DFT computations are performed using PBE functional.

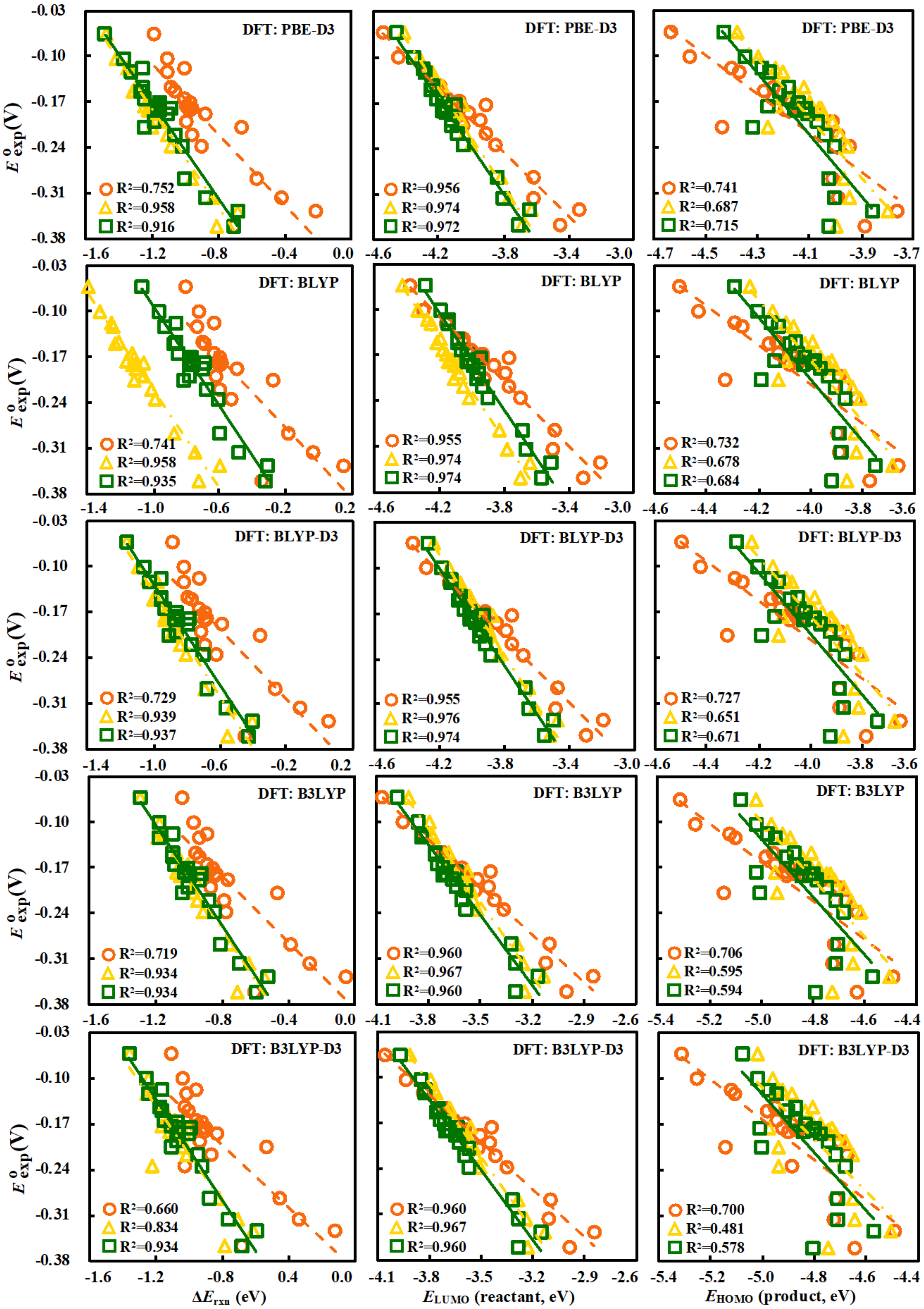

DFT: PBE-D3
DFT: BLYP
DFT: BLYP-D3
DFT: B3LYP
DFT: B3LYP-D3
R²=0.752
R²=0.958
R²=0.916
R²=0.956
R²=0.974
R²=0.972
R²=0.741
R²=0.687
R²=0.715
R²=0.741
R²=0.958
R²=0.935
R²=0.955
R²=0.974
R²=0.974
R²=0.732
R²=0.678
R²=0.684
R²=0.729
R²=0.939
R²=0.937
R²=0.955
R²=0.976
R²=0.974
R²=0.727
R²=0.651
R²=0.671
R²=0.719
R²=0.934
R²=0.934
R²=0.960
R²=0.967
R²=0.960
R²=0.706
R²=0.595
R²=0.594
R²=0.660
R²=0.834
R²=0.934
R²=0.960
R²=0.967
R²=0.960
R²=0.700
R²=0.481
R²=0.578
E°exp(V)
ΔErxn (eV)
ELUMO (reactant, eV)
EHOMO (product, eV)

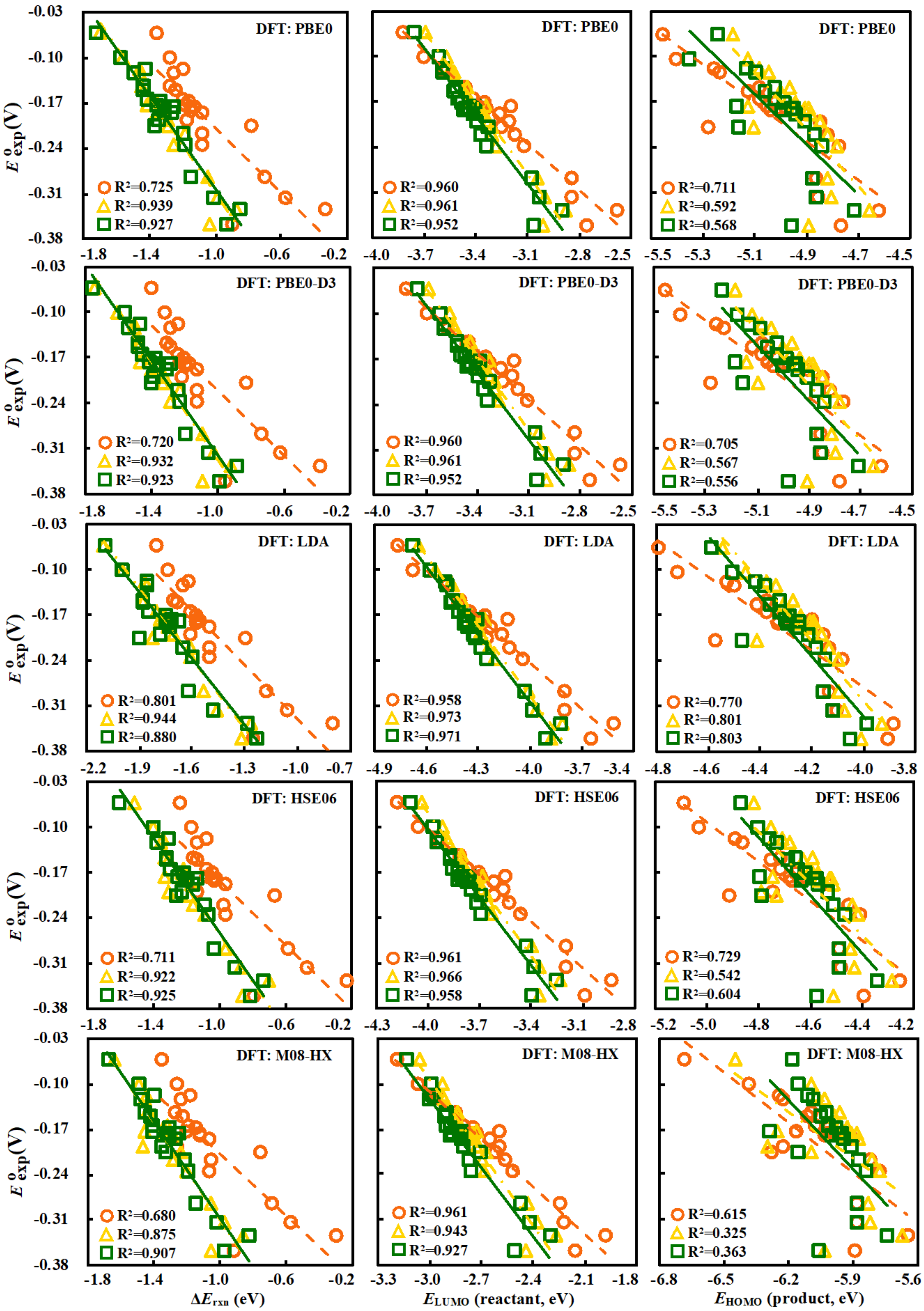
DFT: PBE0
DFT: PBE0-D3
DFT: LDA
DFT: HSE06
DFT: M08-HX
R²=0.725
R²=0.939
R²=0.927
R²=0.960
R²=0.961
R²=0.952
R²=0.711
R²=0.592
R²=0.568
R²=0.720
R²=0.932
R²=0.923
R²=0.960
R²=0.961
R²=0.952
R²=0.705
R²=0.567
R²=0.556
R²=0.801
R²=0.944
R²=0.880
R²=0.958
R²=0.973
R²=0.971
R²=0.770
R²=0.801
R²=0.803
R²=0.711
R²=0.922
R²=0.925
R²=0.961
R²=0.966
R²=0.958
R²=0.729
R²=0.542
R²=0.604
R²=0.680
R²=0.875
R²=0.907
R²=0.961
R²=0.943
R²=0.927
R²=0.615
R²=0.325
R²=0.363
E°exp (V)
ΔErxn (eV)
ELUMO (reactant, eV)
EHOMO (product, eV)

Figure S2. Performance comparisons of exchange-correlation functionals for the prediction of experimentally measured redox potentials, E^{o}_{exp}. The scatter plots in columns from left to right show linear correlations (versus E^{o}_{exp}) of the DFT calculated energy difference between the reactant and product compounds (ΔE_{rxn}), the LUMO energy (E_{LUMO}) of the reactant molecules and the HOMO energy (E_{HOMO}) of the product molecules. The color orange represents both OPT and SPE in gas-phase, the color yellow represents OPT in gas-phase followed by SPE with SOL (including implicit aqueous solvation), and the color green represents both OPT and SPE with SOL.

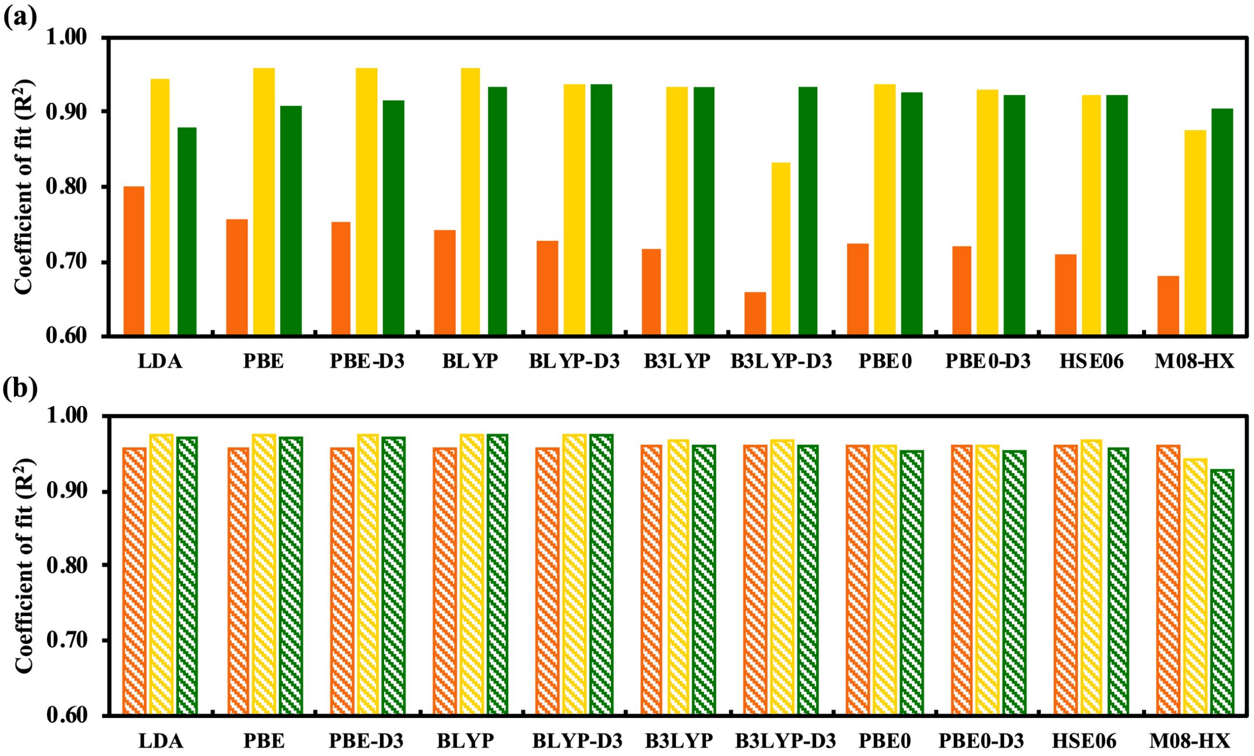

Figure S3. Performance comparisons of exchange-correlation functionals for the prediction of experimentally measured redox potentials, $E^{\mathrm{o}}_{\mathrm{exp}}$. The bar plots **(a)** and **(b)** show R^2 for ΔE_{rxn} and E_{LUMO}, respectively. The color orange represents both OPT and SPE in gas-phase, the color yellow represents OPT in gas-phase followed by SPE with SOL, and the color green represents both OPT and SPE with SOL.

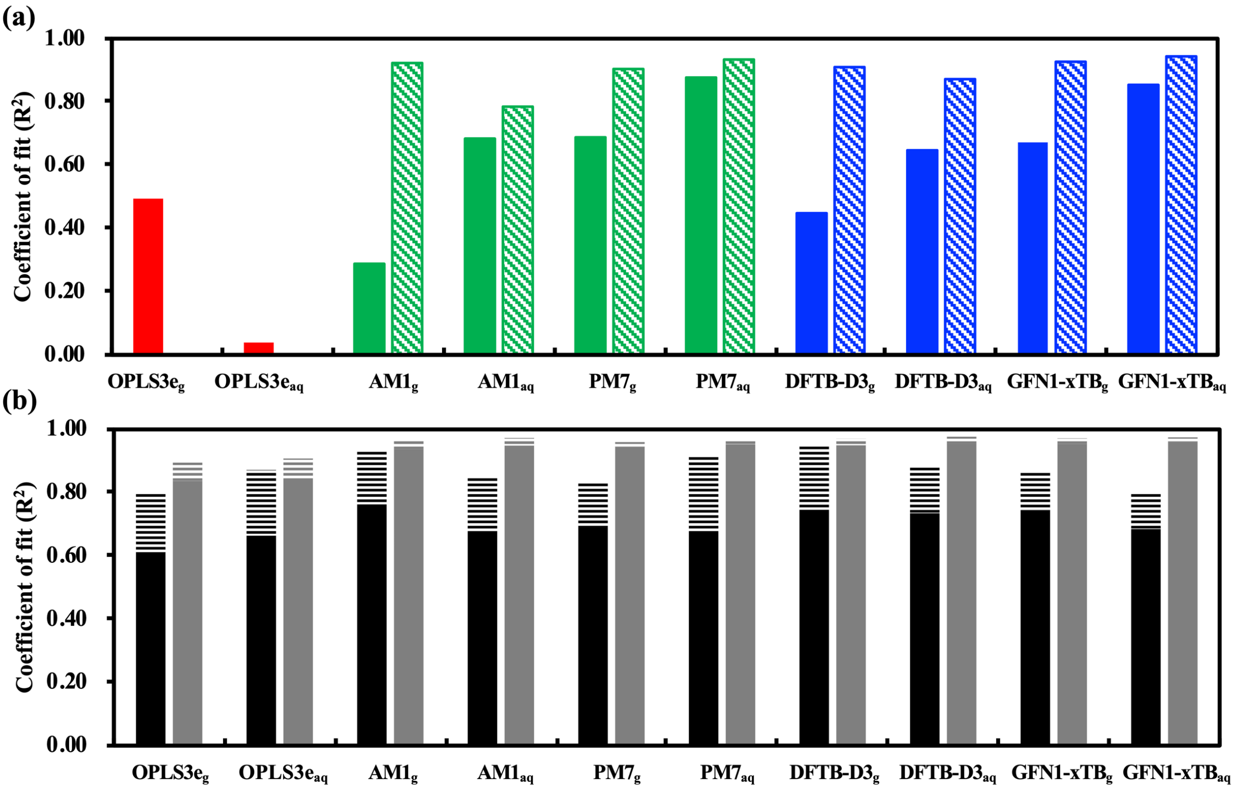

Figure S4. Performance comparisons of low-level methods: FF, SEQM and DFTB. **(a)** shows R^2 for SPE values calculated at these three different levels of theory. Similarly, **(b)** shows R^2 for PBE calculated SPE data on the geometries obtained from these three different levels of theory. In (a) the solid bars show R^2 for SPE when using the ΔE_{rxn} descriptor and the hashed bars show R^2 for SPE when using the E_{LUMO} descriptor. In (b) the solid bars show the SPE results without the implicit solvation effect whereas the hashed bars show the results with the implicit solvation effects taken into account.

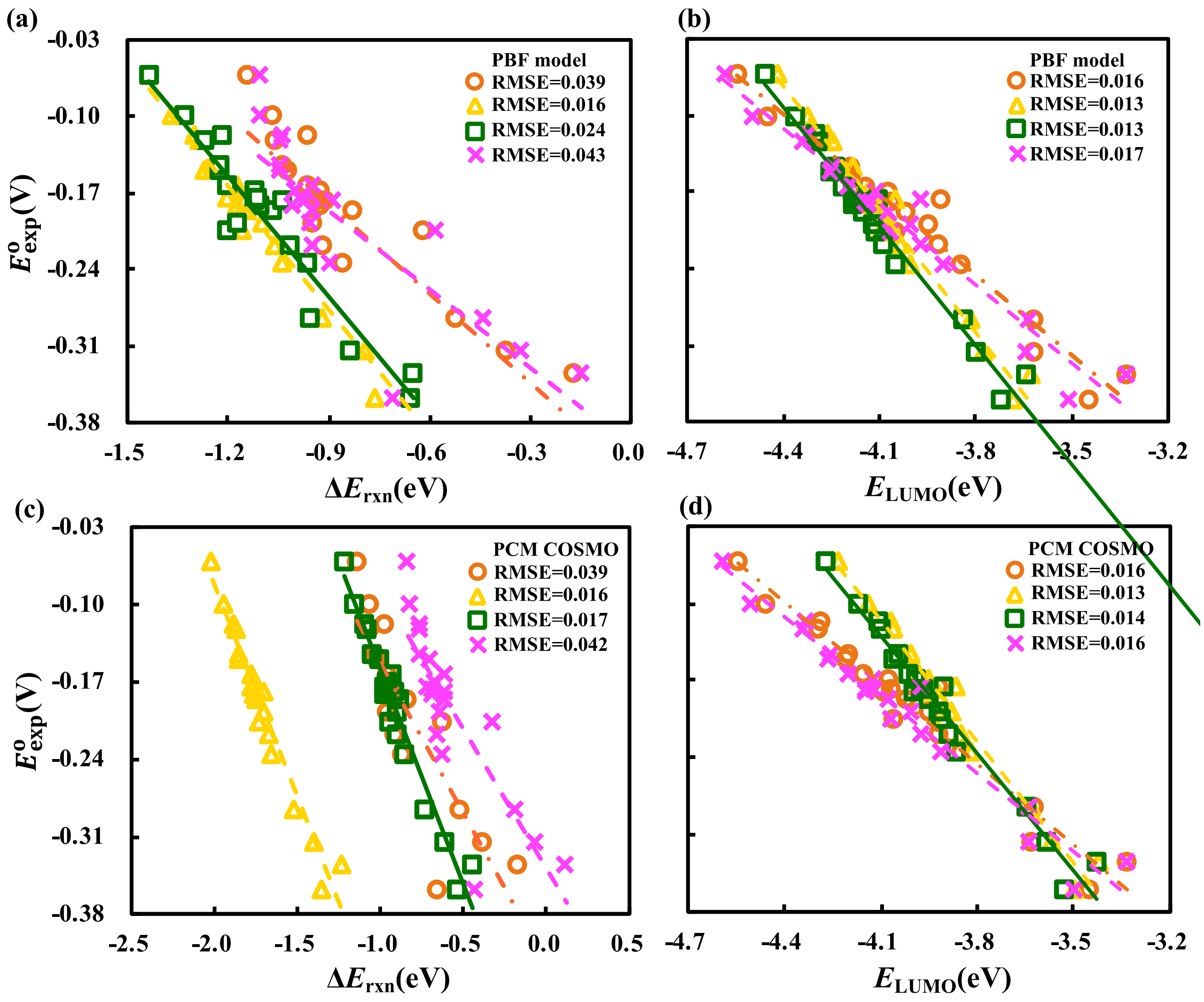

Figure S5. OPT and SPE using two different implicit solvation methods, PBF and PCM (COSMO), respectively. Color orange represents OPT and SPE in gas phase; Color yellow represents OPT in gas phase and SPE in solution phase; Color green represents OPT and SPE in solution phase; Color pink represents OPT in solution phase and SPE in gas phase.

Table S1. A summary of 2D structures, SMILES representations, experimentally measured redox potentials and the predicted redox potentials for 21 alloxazine-based molecules considered in this work. The measured redox potential values have been corrected versus RHE at pH = 7. When analyzing the performance of each descriptor, we divided the set of errors of the 21 molecule into three groups of seven molecules each. The seven molecules with the lowest errors under each approximation are colored yellow, the seven molecules with the highest errors are colored red and the seven in the middle are colored orange. 2D molecule representations have been created using ChemDraw Professional [version 18.0.0.231(4318)] https://www.perkinelmer.com/product/chemdraw-professional-chemdrawpro.

(a) The predicted redox potentials when using ΔE_{rxn} as the descriptor calculated with PBE.

#	Molecule	E^o_{exp} (V)	E^o_g (V)	E^o_s (V)	E^o_{aq} (V)	δE=E^o_g-E^o_{exp}	δE=E^o_s-E^o_{exp}	δE=E^o_{aq}-E^o_{exp}	Ref
**1**	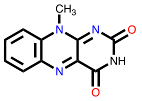 c1cccc(c12)nc3c(n2C)nc(=O)[nH]c3=O	-0.186	-0.200	-0.185	-0.204	-0.014	0.001	-0.018	[1]
**2**	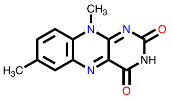 c1cc(C)cc(c12)nc3c(n2C)nc(=O)[nH]c3=O	-0.198	-0.167	-0.202	-0.164	0.031	-0.004	0.034	[1]
**3**	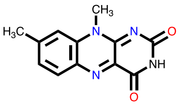 c1c(C)ccc(c12)nc3c(n2C)nc(=O)[nH]c3=O	-0.218	-0.176	-0.217	-0.223	0.042	0.001	-0.005	[1]
**4**	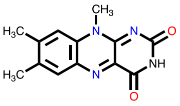 c1c(C)c(C)cc(c12)nc3c(n2C)nc(=O)[nH]c3=O	-0.235	-0.191	-0.225	-0.241	0.044	0.01	-0.006	[1]
**5**	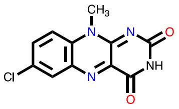	-0.150	-0.147	-0.137	-0.144	0.003	0.013	0.006	[1]

	c1cc(Cl)cc(c12)nc3c(n2C)nc(=O)[nH]c3=O								
**6**	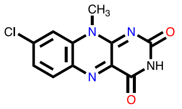 c1c(Cl)ccc(c12)nc3c(n2C)nc(=O)[nH]c3=O	-0.164	-0.163	-0.174	-0.153	0.001	-0.01	0.011	[1]
**7**	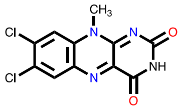 c1c(Cl)c(Cl)cc(c12)nc3c(n2C)nc(=O)[nH]c3=O	-0.117	-0.163	-0.127	-0.150	-0.046	-0.01	-0.033	[1]
**8**	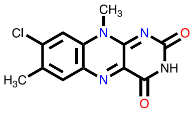 c1c(Cl)c(C)cc(c12)nc3c(n2C)nc(=O)[nH]c3=O	-0.169	-0.172	-0.178	-0.185	-0.003	-0.009	-0.016	[1]
**9**	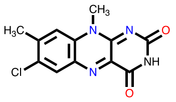 c1c(C)c(Cl)cc(c12)nc3c(n2C)nc(=O)[nH]c3=O	-0.181	-0.173	-0.174	-0.189	0.008	0.007	-0.008	[1]
**10**	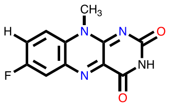 c1cc(F)cc(c12)nc3c(n2C)nc(=O)[nH]c3=O	-0.145	-0.143	-0.145	-0.147	0.002	0	-0.002	[1]
**11**	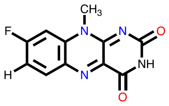 c1c(F)ccc(c12)nc3c(n2C)nc(=O)[nH]c3=O	-0.178	-0.177	-0.202	-0.185	0.001	-0.024	-0.007	[1]
**12**	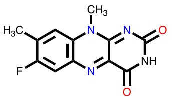 c1c(C)c(F)cc(c12)nc3c(n2C)nc(=O)[nH]c3=O	-0.177	-0.171	-0.182	-0.214	0.006	-0.005	-0.037	[1]

**13**	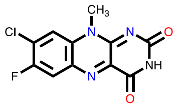 c1c(Cl)c(F)cc(c12)nc3c(n2C)nc(=O)[nH]c3=O	-0.123	-0.137	-0.132	-0.128	-0.014	-0.009	-0.005	[1]
**14**	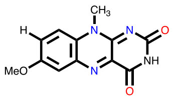 COc(c1)ccc(c12)n(C)c3c(n2)c(=O)[nH]c(n3)=O	-0.175	-0.169	-0.163	-0.187	0.006	0.012	-0.012	[1]
**15**	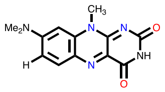 c1cc(N(C)C)cc(c12)n(C)c3c(n2)c(=O)[nH]c(n3)=O	-0.359	-0.246	-0.328	-0.355	0.113	0.031	0.004	[1]
**16**	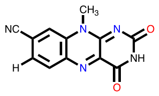 c1cc(C#N)cc(c12)n(C)c3c(n2)c(=O)[nH]c(n3)=O	-0.062	-0.115	-0.075	-0.068	-0.053	-0.013	-0.006	[1]
**17**	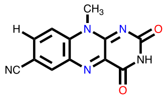 N#Cc(c1)ccc(c12)n(C)c3c(n2)c(=O)[nH]c(n3)=O	-0.100	-0.135	-0.100	-0.108	-0.035	0	-0.008	[1]
**18**	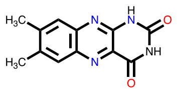 c1c(C)c(C)cc(c12)nc3c(n2)c(=O)[nH]c(=O)[nH]3	-0.286	-0.283	-0.270	-0.243	0.003	0.016	0.043	[2]
**19**	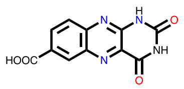	-0.206	-0.257	-0.180	-0.152	-0.051	0.026	0.054	[2]

	O=C(O)c(c1)ccc(c12)nc3c(n2)c(=O)[nH]c(=O)[nH]3								
**20**	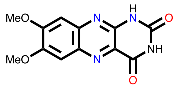 COc(c1)c(OC)cc(c12)nc3c(n2)c(=O)[nH]c(=O)[nH]3	-0.336	-0.378	-0.373	-0.358	-0.042	-0.037	-0.022	[2]
**21**	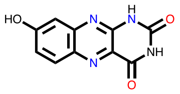 c1cc(O)cc(c12)nc3c(n2)c(=O)[nH]c(=O)[nH]3	-0.316	-0.323	-0.319	-0.289	-0.007	-0.003	0.027	[2]
**MAE**						0.025	0.011	0.017	
**Maximum signed error [Molecule #]**						0.113 [15]	-0.037 [20]	0.054 [19]	

(b) The predicted redox potentials when using E_{LUMO} as the descriptor calculated with PBE.

#	Molecule	E^{o}_{exp} (V)	E^{o}_{g} (V)	E^{o}_{s} (V)	E^{o}_{aq} (V)	$\delta E=E^{o}_{g}-E^{o}_{exp}$	$\delta E=E^{o}_{s}-E^{o}_{exp}$	$\delta E=E^{o}_{aq}-E^{o}_{exp}$	Ref
**1**	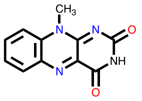 c1cccc(c12)nc3c(n2C)nc(=O)[nH]c3=O	-0.186	-0.192	-0.185	-0.182	-0.006	0.001	0.004	[1]
**2**	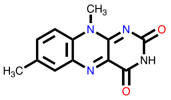 c1cc(C)cc(c12)nc3c(n2C)nc(=O)[nH]c3=O	-0.198	-0.209	-0.197	-0.193	-0.011	0.001	0.005	[1]
**3**	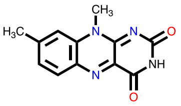 c1c(C)ccc(c12)nc3c(n2C)nc(=O)[nH]c3=O	-0.218	-0.217	-0.208	-0.205	0.001	0.01	0.013	[1]

**4**	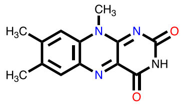 c1c(C)c(C)cc(c12)nc3c(n2C)nc(=O)[nH]c3=O	-0.235	-0.234	-0.220	-0.218	0.001	0.015	0.017	[1]
**5**	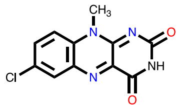 c1cc(Cl)cc(c12)nc3c(n2C)nc(=O)[nH]c3=O	-0.150	-0.147	-0.146	-0.146	0.003	0.004	0.004	[1]
**6**	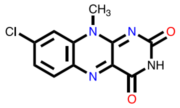 c1c(Cl)ccc(c12)nc3c(n2C)nc(=O)[nH]c3=O	-0.164	-0.160	-0.158	-0.158	0.004	0.006	0.006	[1]
**7**	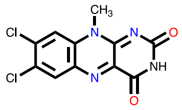 c1c(Cl)c(Cl)cc(c12)nc3c(n2C)nc(=O)[nH]c3=O	-0.117	-0.127	-0.125	-0.128	-0.01	-0.008	-0.011	[1]
**8**	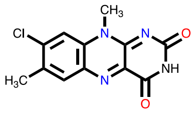 c1c(Cl)c(C)cc(c12)nc3c(n2C)nc(=O)[nH]c3=O	-0.169	-0.179	-0.170	-0.171	-0.01	-0.001	-0.002	[1]
**9**	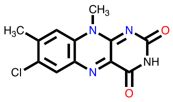 c1c(C)c(Cl)cc(c12)nc3c(n2C)nc(=O)[nH]c3=O	-0.181	-0.174	-0.170	-0.170	0.007	0.011	0.011	[1]
**10**	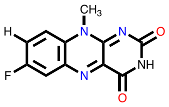 c1cc(F)cc(c12)nc3c(n2C)nc(=O)[nH]c3=O	-0.145	-0.149	-0.154	-0.152	-0.004	-0.009	-0.007	[1]

**11**	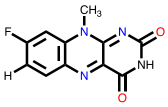 c1c(F)ccc(c12)nc3c(n2C)nc(=O)[nH]c3=O	-0.178	-0.174	-0.179	-0.176	0.004	-0.001	0.002	[1]
**12**	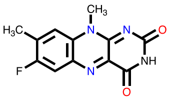 c1c(C)c(F)cc(c12)nc3c(n2C)nc(=O)[nH]c3=O	-0.177	-0.180	-0.181	-0.178	-0.003	-0.004	-0.001	[1]
**13**	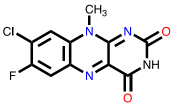 c1c(Cl)c(F)cc(c12)nc3c(n2C)nc(=O)[nH]c3=O	-0.123	-0.126	-0.131	-0.131	-0.003	-0.008	-0.008	[1]
**14**	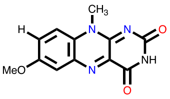 COc(c1)ccc(c12)n(C)c3c(n2)c(=O)[nH]c(n3)=O	-0.175	-0.218	-0.204	-0.199	-0.043	-0.029	-0.024	[1]
**15**	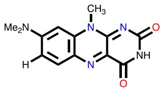 c1cc(N(C)C)cc(c12)n(C)c3c(n2)c(=O)[nH]c(n3)=O	-0.359	-0.331	-0.341	-0.339	0.028	0.018	0.02	[1]
**16**	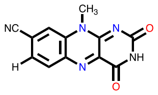 c1cc(C#N)cc(c12)n(C)c3c(n2)c(=O)[nH]c(n3)=O	-0.062	-0.064	-0.066	-0.071	-0.002	-0.004	-0.009	[1]
**17**	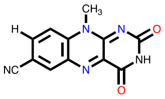	-0.100	-0.085	-0.102	-0.105	0.015	-0.002	-0.005	[1]

	N#Cc(c1)ccc(c12)n(C)c3c(n2)c(=O)[nH]c(n3)=O								
**18**	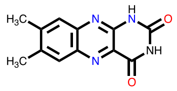 c1c(C)c(C)cc(c12)nc3c(n2)c(=O)[nH]c(=O)[nH]3	-0.286	-0.290	-0.292	-0.296	-0.004	-0.006	-0.01	[2]
**19**	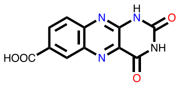 O=C(O)c(c1)ccc(c12)nc3c(n2)c(=O)[nH]c(=O)[nH]3	-0.206	-0.183	-0.187	-0.195	0.023	0.019	0.011	[2]
**20**	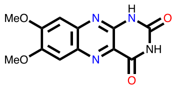 COc(c1)c(OC)cc(c12)nc3c(n2)c(=O)[nH]c(=O)[nH]3	-0.336	-0.360	-0.362	-0.367	-0.024	-0.026	-0.031	[2]
**21**	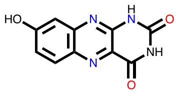 c1cc(O)cc(c12)nc3c(n2)c(=O)[nH]c(=O)[nH]3	-0.316	-0.289	-0.307	-0.309	0.027	0.009	0.007	[2]
**MAE**						0.011	0.009	0.010	
**Maximum signed error [Molecule #]**						-0.043 [14]	-0.029 [14]	-0.031 [20]	

Table S2. Performance comparisons of the exchange-correlation functionals for the prediction of the experimentally measured redox potentials. DFT_g represents both OPT and SPE in gas phase; DFT_s represents OPT in gas-phase followed by SPE in SOL; DFT_{aq} represents both OPT and SPE in SOL.

(a) R^2 and RMSE when using ΔE_{rxn} as the descriptor

Scheme for OPT and SPE	DFT_g		DFT_s		DFT_{aq}	
DFT methods	R^2	RMSE (V)	R^2	RMSE (V)	R^2	RMSE (V)
LDA	0.801	0.035	0.944	0.019	0.880	0.027
PBE	0.756	0.039	0.959	0.016	0.910	0.024
PBE-D3	0.752	0.039	0.958	0.016	0.916	0.023
BLYP	0.741	0.040	0.958	0.016	0.935	0.020
BLYP-D3	0.729	0.041	0.939	0.019	0.937	0.020
B3LYP	0.719	0.042	0.934	0.020	0.934	0.020
B3LYP-D3	0.660	0.046	0.834	0.032	0.934	0.020
PBE0	0.725	0.041	0.939	0.019	0.927	0.021
PBE0-D3	0.720	0.042	0.932	0.020	0.923	0.022
HSE06	0.711	0.042	0.922	0.022	0.925	0.022
M08-HX	0.680	0.044	0.875	0.028	0.907	0.024

(b) R^2 and RMSE when using E_{LUMO} as the descriptor

Scheme for OPT and SPE	DFT_g		DFT_s		DFT_{aq}	
DFT methods	R^2	RMSE (V)	R^2	RMSE (V)	R^2	RMSE (V)
LDA	0.958	0.016	0.973	0.013	0.971	0.013
PBE	0.956	0.016	0.974	0.013	0.972	0.013
PBE-D3	0.956	0.016	0.974	0.013	0.972	0.013
BLYP	0.955	0.017	0.974	0.013	0.974	0.013
BLYP-D3	0.955	0.017	0.976	0.012	0.974	0.013
B3LYP	0.960	0.016	0.967	0.014	0.960	0.016
B3LYP-D3	0.960	0.016	0.967	0.014	0.960	0.016
PBE0	0.960	0.016	0.961	0.015	0.952	0.017
PBE0-D3	0.960	0.016	0.961	0.016	0.952	0.017
HSE06	0.961	0.015	0.966	0.015	0.958	0.016
M08-HX	0.961	0.015	0.943	0.019	0.927	0.021

(c) R^2 and RMSE when using E_{HOMO} as the descriptor

Scheme for OPT and SPE	DFT_g		DFT_s		DFT_{aq}	
DFT methods	R^2	RMSE (V)	R^2	RMSE (V)	R^2	RMSE (V)
LDA	0.770	0.038	0.801	0.035	0.803	0.035
PBE	0.743	0.040	0.700	0.043	0.731	0.041
PBE-D3	0.741	0.040	0.687	0.044	0.715	0.042
BLYP	0.732	0.041	0.678	0.045	0.684	0.044
BLYP-D3	0.727	0.041	0.651	0.046	0.671	0.045
B3LYP	0.706	0.043	0.595	0.050	0.594	0.050
B3LYP-D3	0.700	0.043	0.481	0.057	0.578	0.051
PBE0	0.711	0.042	0.592	0.050	0.568	0.052
PBE0-D3	0.705	0.043	0.567	0.052	0.556	0.052
HSE06	0.729	0.041	0.542	0.053	0.604	0.049
M08-HX	0.615	0.049	0.325	0.064	0.363	0.063

Table S3. Performance comparisons of nine different SEQM methods for the prediction of experimentally measured redox potentials. $SEQM_g$ represents OPT or SPE in gas-phase. PBE_g and $B3LYP_g$ represent SPE calculation in gas-phase with the two respective functionals. PBE_s and $B3LYP_s$ represent SPE calculation in SOL and with the two respective functionals.

(a) R^2 and RMSE when using ΔE_{rxn} as the descriptor

Scheme for OPT and SPE	OPT:$SEQM_g$ SPE:$SEQM_g$		OPT:$SEQM_g$ SPE:PBE_g		OPT:$SEQM_g$ SPE:PBE_s		OPT: $SEQM_g$ SPE: $B3LYP_g$		OPT: $SEQM_g$ SPE: $B3LYP_s$	
Method	R^2	RMSE(V)	R^2	RMSE(V)	R^2	RMSE(V)	R^2	RMSE(V)	R^2	RMSE(V)
AM1	0.288	0.066	0.763	0.038	0.932	0.020	0.707	0.043	0.933	0.020
MNDO	0.100	0.074	0.630	0.048	0.823	0.033	0.576	0.051	0.818	0.034
MNDOD	0.206	0.070	0.650	0.046	0.845	0.031	0.595	0.050	0.830	0.032
PM3	0.502	0.055	0.737	0.040	0.946	0.018	0.683	0.044	0.919	0.022
PM6	0.598	0.050	0.773	0.037	0.866	0.029	0.727	0.041	0.881	0.027
PM6-D3	0.598	0.050	0.778	0.037	0.914	0.023	0.722	0.041	0.918	0.022
PM6-D3H4X	0.591	0.050	0.753	0.039	0.821	0.033	0.710	0.042	0.842	0.031
PM7	0.688	0.044	0.691	0.044	0.834	0.032	0.666	0.045	0.837	0.032
RM1	0.523	0.054	0.816	0.034	0.954	0.017	0.816	0.034	0.976	0.012

(b) R^2 and RMSE when using E_{LUMO} as the descriptor

Scheme for OPT and SPE	OPT:$SEQM_g$ SPE:$SEQM_g$		OPT:$SEQM_g$ SPE:PBE_g		OPT:$SEQM_g$ SPE:PBE_s		OPT: $SEQM_g$ SPE: $B3LYP_g$		OPT: $SEQM_g$ SPE: $B3LYP_s$	
Method	R^2	RMSE(V)	R^2	RMSE(V)	R^2	RMSE(V)	R^2	RMSE(V)	R^2	RMSE(V)
AM1	0.923	0.022	0.938	0.020	0.965	0.015	0.947	0.018	0.963	0.015
MNDO	0.874	0.028	0.936	0.020	0.962	0.015	0.947	0.018	0.961	0.016
MNDOD	0.857	0.030	0.938	0.020	0.963	0.015	0.948	0.018	0.960	0.016
PM3	0.875	0.028	0.918	0.023	0.945	0.018	0.926	0.021	0.936	0.020
PM6	0.903	0.024	0.933	0.020	0.954	0.017	0.942	0.019	0.954	0.017
PM6-D3	0.903	0.025	0.934	0.020	0.955	0.017	0.942	0.019	0.955	0.017
PM6-D3H4X	0.907	0.024	0.934	0.020	0.953	0.017	0.945	0.018	0.960	0.016
PM7	0.904	0.024	0.945	0.018	0.960	0.016	0.955	0.017	0.962	0.015
RM1	0.919	0.022	0.940	0.019	0.962	0.015	0.940	0.019	0.966	0.014

Table S4. Performance comparisons of nine different SEQM methods for prediction of experimentally measured redox potentials. $SEQM_{aq}$ represents OPT or SPE in SOL. PBE_g and $B3LYP_g$ represent SPE calculation in gas-phase with the two respective functionals. PBE_s and $B3LYP_s$ represent SPE calculation in SOL and with the two respective functionals.

(a) R^2 and RMSE when using ΔE_{rxn} as the descriptor

Scheme for OPT and SPE	OPT:$SEQM_{aq}$ SPE:$SEQM_{aq}$		OPT:$SEQM_{aq}$ SPE:PBE_g		OPT:$SEQM_{aq}$ SPE:PBE_s		OPT: $SEQM_{aq}$ SPE: $B3LYP_g$		OPT: $SEQM_{aq}$ SPE: $B3LYP_s$	
Method	R^2	RMSE(V)	R^2	RMSE(V)	R^2	RMSE(V)	R^2	RMSE(V)	R^2	RMSE(V)
AM1	0.684	0.044	0.676	0.045	0.850	0.030	0.656	0.046	0.846	0.031
MNDO	0.448	0.058	0.490	0.056	0.803	0.035	0.477	0.057	0.812	0.034
MNDOD	0.424	0.060	0.489	0.056	0.817	0.034	0.470	0.057	0.819	0.033
PM3	0.678	0.045	0.789	0.036	0.892	0.026	0.765	0.038	0.928	0.021
PM6	0.820	0.033	0.712	0.042	0.928	0.021	0.673	0.045	0.927	0.021
PM6-D3	0.817	0.034	0.699	0.043	0.914	0.023	0.660	0.046	0.911	0.023
PM6-D3H4X	0.801	0.035	0.678	0.045	0.855	0.030	0.634	0.048	0.851	0.030
PM7	0.877	0.028	0.678	0.045	0.917	0.023	0.632	0.048	0.895	0.025
RM1	0.804	0.035	0.692	0.044	0.855	0.030	0.662	0.046	0.881	0.027

(b) R^2 and RMSE when using E_{LUMO} as the descriptor

Scheme for OPT and SPE	OPT:$SEQM_{aq}$ SPE:$SEQM_{aq}$		OPT:$SEQM_{aq}$ SPE:PBE_g		OPT:$SEQM_{aq}$ SPE:PBE_s		OPT: $SEQM_{aq}$ SPE: $B3LYP_g$		OPT: $SEQM_{aq}$ SPE: $B3LYP_s$	
Method	R^2	RMSE(V)	R^2	RMSE(V)	R^2	RMSE(V)	R^2	RMSE(V)	R^2	RMSE(V)
AM1	0.785	0.036	0.949	0.018	0.973	0.013	0.957	0.016	0.967	0.014
MNDO	0.660	0.046	0.966	0.014	0.976	0.012	0.958	0.016	0.942	0.019
MNDOD	0.668	0.045	0.965	0.015	0.975	0.012	0.958	0.016	0.941	0.019
PM3	0.700	0.043	0.936	0.020	0.951	0.017	0.938	0.020	0.928	0.021
PM6	0.927	0.021	0.944	0.019	0.961	0.016	0.943	0.019	0.951	0.017
PM6-D3	0.927	0.021	0.944	0.019	0.960	0.016	0.944	0.019	0.951	0.017
PM6-D3H4X	0.929	0.021	0.943	0.019	0.959	0.016	0.942	0.019	0.951	0.017
PM7	0.934	0.020	0.953	0.017	0.965	0.015	0.955	0.017	0.951	0.017
RM1	0.839	0.031	0.955	0.017	0.967	0.014	0.961	0.016	0.963	0.015

Table S5. Performance comparisons of FF(OPLS3e) in combination with the different DFT methods for the prediction of experimentally measured redox potentials. FF_g represents OPT or SPE in gas-phase. FF_{aq} represents OPT or SPE in SOL. PBE_g and $B3LYP_g$, represent SPE calculation in gas-phase with the two respective functionals. PBE_s and $B3LYP_s$ represent SPE calculation in SOL and with the two respective functionals.

(a) R^2 and RMSE when using ΔE_{rxn} as the descriptor

Scheme for OPT and SPE	OPT:FF_g SPE:FF_g		OPT: FF_g SPE: PBE_g		OPT: FF_g SPE: PBE_s		OPT: FF_g SPE: $B3LYP_g$		OPT: FF_g SPE: $B3LYP_s$	
Method	R^2	RMSE(V)	R^2	RMSE(V)	R^2	RMSE(V)	R^2	RMSE(V)	R^2	RMSE(V)
OPLS3e	0.494	0.056	0.612	0.049	0.798	0.035	0.647	0.047	0.767	0.038
Scheme for OPT and SPE	OPT:FF_{aq} SPE:FF_{aq}		OPT: FF_{aq} SPE: PBE_g		OPT: FF_{aq} SPE: PBE_s		OPT: FF_{aq} SPE: $B3LYP_g$		OPT: FF_{aq} SPE: $B3LYP_s$	
Method	R^2	RMSE(V)	R^2	RMSE(V)	R^2	RMSE(V)	R^2	RMSE(V)	R^2	RMSE(V)
OPLS3e	0.039	0.077	0.664	0.046	0.872	0.028	0.644	0.047	0.815	0.034

(b) R^2 and RMSE when using E_{LUMO} as the descriptor

Scheme for OPT and SPE	OPT: FF_g SPE: PBE_g		OPT: FF_g SPE: PBE_s		OPT: FF_g SPE: $B3LYP_g$		OPT: FF_g SPE: $B3LYP_s$	
Method	R^2	RMSE(V)	R^2	RMSE(V)	R^2	RMSE(V)	R^2	RMSE(V)
OPLS3e	0.837	0.032	0.895	0.025	0.847	0.031	0.914	0.023
Scheme for OPT and SPE	OPT: FF_{aq} SPE: PBE_{aq}		OPT: FF_{aq} SPE: PBE_s		OPT: FF_{aq} SPE: $B3LYP_g$		OPT: FF_{aq} SPE: $B3LYP_s$	
Method	R^2	RMSE(V)	R^2	RMSE(V)	R^2	RMSE(V)	R^2	RMSE(V)
OPLS3e	0.845	0.031	0.908	0.024	0.855	0.030	0.925	0.021

Table S6. Performance comparisons of the two different DFTB methods for the prediction of experimentally measured redox potentials. $DFTB_g$ represents OPT or SPE in gas-phase. PBE_g and $B3LYP_g$ represent SPE calculation in gas-phase with the two respective functionals. PBE_s and $B3LYP_s$ represent SPE calculation in SOL and with the two respective functionals.

(a) R^2 and RMSE when using ΔE_{rxn} as the descriptor

Scheme for OPT and SPE	OPT:$DFTB_g$ SPE:$DFTB_g$		OPT: $DFTB_g$ SPE: PBE_g		OPT: $DFTB_g$ SPE: PBE_s		OPT: $DFTB_g$ SPE: $B3LYP_g$		OPT: $DFTB_g$ SPE: $B3LYP_s$	
Method	R^2	RMSE(V)	R^2	RMSE(V)	R^2	RMSE(V)	R^2	RMSE(V)	R^2	RMSE(V)
DFTB-D3	0.448	0.058	0.746	0.040	0.946	0.018	0.715	0.042	0.925	0.021
GFN1-xTB	0.672	0.045	0.744	0.040	0.863	0.029	0.704	0.043	0.854	0.030

(b) R^2 and RMSE when using E_{LUMO} as the descriptor

Scheme for OPT and SPE	OPT:$DFTB_g$ SPE:$DFTB_g$		OPT: $DFTB_g$ SPE: PBE_g		OPT: $DFTB_g$ SPE: PBE_s		OPT: $DFTB_g$ SPE: $B3LYP_g$		OPT: $DFTB_g$ SPE: $B3LYP_s$	
Method	R^2	RMSE(V)	R^2	RMSE(V)	R^2	RMSE(V)	R^2	RMSE(V)	R^2	RMSE(V)
DFTB-D3	0.910	0.024	0.950	0.018	0.971	0.013	0.947	0.018	0.948	0.018
GFN1-xTB	0.927	0.021	0.954	0.017	0.972	0.013	0.960	0.016	0.972	0.013

Table S7. Performance comparisons of two different DFTB methods for the prediction of experimentally measured redox potentials. $DFTB_{aq}$ represents OPT or SPE in SOL. PBE_g and $B3LYP_g$ represent SPE calculation in gas-phase with the two respective functionals. PBE_s and $B3LYP_s$ represent SPE calculation in SOL and with the two respective functionals.

(a) R^2 and RMSE when using ΔE_{rxn} as the descriptor

Scheme for OPT and SPE	OPT:$DFTB_{aq}$ SPE:$DFTB_{aq}$		OPT: $DFTB_{aq}$ SPE: PBE_g		OPT: $DFTB_{aq}$ SPE: PBE_s		OPT: $DFTB_{aq}$ SPE: $B3LYP_g$		OPT: $DFTB_{aq}$ SPE: $B3LYP_s$	
Method	R^2	RMSE(V)	R^2	RMSE(V)	R^2	RMSE(V)	R^2	RMSE(V)	R^2	RMSE(V)
DFTB-D3	0.647	0.047	0.735	0.040	0.881	0.027	0.703	0.043	0.885	0.027
GFN1-xTB	0.854	0.030	0.685	0.044	0.801	0.035	0.642	0.047	0.811	0.034

(b) R^2 and RMSE when using E_{LUMO} as the descriptor

Scheme for OPT and SPE	OPT:$DFTB_{aq}$ SPE:$DFTB_{aq}$		OPT: $DFTB_{aq}$ SPE: PBE_g		OPT: $DFTB_{aq}$ SPE: PBE_s		OPT: $DFTB_{aq}$ SPE: $B3LYP_g$		OPT: $DFTB_{aq}$ SPE: $B3LYP_s$	
Method	R^2	RMSE(V)	R^2	RMSE(V)	R^2	RMSE(V)	R^2	RMSE(V)	R^2	RMSE(V)
DFTB-D3	0.872	0.028	0.955	0.017	0.977	0.012	0.951	0.017	0.961	0.016
GFN1-xTB	0.944	0.019	0.961	0.016	0.975	0.012	0.962	0.015	0.971	0.013

DFT calculation details

As DFT options in Jaguar, a medium grid density for OPT and a fine grid density for SPE calculations have been used. Energy and RMS density matrix change convergence criteria are set to the default values of 5.0×10^{-5} and 5.0×10^{-6} Hartree, respectively. The direct inversion in the iterative subspace is employed as the convergence scheme. For OPT, we used Jaguar's mixed pseudospectral grids with default cutoffs. For SPE calculations, we used pseudospectral grids with accurate cutoffs. To treat the solvated molecules in water, we used the PBF solver.[3,4] The calculations are performed with LACVP^{**++} basis set with polarization and diffuse functions.[5,6] The LACVP basis set includes effective core potentials (ECP), which represent the core electrons in a parametrized form. Therefore, ECPs speed up calculations meanwhile enabling calculations on heavy elements at similar computing costs as for the calculations on light elements. We note that for the elements from H to Ar, such as in the current work, the widely employed 6-31G basis and the LACVP are essentially the same. The alloxazines considered in this work contain C, H, O, N, F and Cl atoms, and thus, the use of LACVP^{**++} basis in this work is consistent with a 6-31G^{**++} basis.

References for Supplementary Information

1. Hasford, J. J. & Rizzo, C. J. Linear free energy substitutent effect on flavin redox chemistry. *J. Am. Chem. Soc.* **120**, 2251–2255 (1998).
2. Lin, K. *et al.* A redox-flow battery with an alloxazine-based organic electrolyte. *Nat. Energy* **1**, 16102 (2016).
3. Tannor, D. J. *et al.* Accurate First Principles Calculation of Molecular Charge Distributions and Solvation Energies from Ab Initio Quantum Mechanics and Continuum Dielectric Theory. *J. Am. Chem. Soc.* **116**, 11875–11882 (1994).
4. Bochevarov, A. D. *et al.* Jaguar: A high-performance quantum chemistry software program with strengths in life and materials sciences. *Int. J. Quantum Chem.* **113**, 2110–2142 (2013)
5. Hay, P. J. & Wadt, W. R. Ab Initio Effective Core Potentials for Molecular Calculations. Potentials. for K to Au Including the Outermost Core Orbitale. *J. Chem. Phys.* **82**, 299–310 (1985).
6. Jaguar, version 10.5. Schrödinger, Inc., New York, NY, 2019.
